# ER stress in D1-MSNs mediates cocaine-induced behavioral plasticity *via* the ATF4–SPTLC1 axis

**DOI:** 10.3389/fphar.2025.1677343

**Published:** 2025-11-25

**Authors:** Yue Zhao, Shu Li, Linhong Jiang, Ying Zhao, Weihong Kuang, Qingfan Wei, Yuanyi Zhou, Shuang Han, Liang Wang, Hongchun Li, Yanping Dai, Xiaofeng Yang, Siqi Xu, Feng Qin, Rong Chen, Yaxing Chen, Chunqi Liu, Qian Bu, Bo Chen, Bin Liu, Meng Qin, Yinglan Zhao, Xiaobo Cen

**Affiliations:** 1 Mental Health Center and Center for Preclinical Safety Evaluation of Drugs, State Key Laboratory of Biotherapy, West China Hospital, Sichuan University, Chengdu, China; 2 Department of Precision Medicine Center, West China Hospital, Sichuan University, Chengdu, Sichuan, China; 3 Chengdu Westchina Frontier Pharmatech, Co., Ltd., Chengdu, China

**Keywords:** cocaine, D1-type medium spiny neurons, endoplasmic reticulum stress, SPTLC1, sphingolipid metabolism

## Abstract

**Background:**

Cocaine-induced endoplasmic reticulum (ER) stress has been increasingly recognized, but its neuronal specificity and functional significance remain unclear. Because the ER is also a major site for lipid and sphingolipid biosynthesis, cocaine-triggered ER stress may influence metabolic pathways linked to cellular stress signaling. Here, we sought to define the cell-type specificity and downstream consequences of cocaine-induced ER stress in the nucleus accumbens (NAc).

**Methods:**

We combined cocaine administration with ultrastructural analysis of ER morphology, immunohistochemical and molecular profiling of ER stress pathways, and assays of sphingolipid biosynthesis in the NAc. We also evaluated the effects of pharmacological inhibition of ER stress and sphingolipid synthesis, and performed D1-MSN–specific knockdown of *Atf4* and *Sptlc1* to assess their contributions to cocaine-induced behavioral and neuroplastic adaptations.

**Results:**

Cocaine selectively activated ER stress in dopamine receptor 1 (D1)–expressing medium spiny neurons (MSNs), marked by induction of activating transcription factor 4 (ATF4). Cocaine also upregulated serine palmitoyltransferase long-chain base subunit 1 (SPTLC1), and promoter analysis with functional validation identified *Sptlc1* as a direct target of ATF4. ATF4 activation was thus coupled to remodeling of sphingolipid metabolism. Blocking ER stress or sphingolipid synthesis–and D1-MSN–specific knockdown of *Atf4* or *Sptlc1*—markedly reduced cocaine-induced behavioral and neuroplastic changes.

**Discussion:**

These findings identify a D1-MSN ER stress response that promotes cocaine-induced neuroadaptations via the ATF4–SPTLC1 signaling axis and suggest a potential therapeutic target for cocaine addiction.

## 1 Introduction

Substance use disorders (SUDs) are chronic and relapsing conditions characterized by compulsive drug-seeking behavior despite the harmful consequences of drug use (Luscher et al., 2020; Wang H. et al., 2018). Exposure to addictive substances triggers rapid release of dopamine, forming multiple widely recognized brain signals and circuits that mediate reward and reinforcement (Kuhar et al., 1991; Wise and Robble, 2020). Mechanistic studies of SUDs have primarily focused on elucidating molecular and cellular events in neuronal cells, such as gene transcriptional regulation, intracellular signaling, and synaptic receptors alterations (Nestler and Lüscher, 2019; Russo et al., 2010; Robison and Nestler, 2011; Teague and Nestler, 2022; Zhao et al., 2022). In recent years, an increasing number of studies show that organelle stress, such as endoplasmic reticulum (ER) stress, is closely associated with drug exposure (Go et al., 2017; Thornton et al., 2021) and neuroplastic changes (Cole et al., 2021). Nevertheless, how ER stress and its integrated signaling networks regulate cocaine-induced neurobehaviors remains largely unknown.

The ER is one of the most metabolically active organelles that plays a central role in the maintenance of metabolic homeostasis such as protein and lipid synthesis (Dixon and Trimmer, 2023). Since neurons have a high demand for synaptic and calcium signaling, the ER is vital for supporting synaptic plasticity and neuronal excitability (Martínez et al., 2018; Verkhratsky, 2005). Various stress circumstances can cause a condition called ER stress, which leads to the accumulation of unfolded or misfolded proteins (Hetz and Saxena, 2017). In turn, a sophisticated and highly dynamic signaling network referred to as the unfolded protein response (UPR) is initiated to manage ER stress and facilitate stress adaptation (Marciniak et al., 2022). ER stress is increasingly recognized as a significant contributor to neurodegenerative diseases and psychiatric disorders (Yang et al., 2024; Pavlovsky et al., 2013). Progressive ER stress in the brain occurs during exposure to addictive drugs (Fauzi et al., 2022; Chen et al., 2021; Jayanthi et al., 2009; Shin et al., 2007; Wang Y. et al., 2018); several animal studies have shown that exposure to psychostimulants can induce ER stress in the dorsal striatum and hippocampus (Go et al., 2010; Wen et al., 2019; Yang et al., 2020). Cocaine-induced UPR in glia or striatal neurons may lead to neuropathological features, such as inflammation or neuronal death (Guo et al., 2015; Periyasamy et al., 2016). Despite these advances, direct evidence demonstrating the role of ER stress, especially the principal transcriptional branches of UPR, in regulating the effect of cocaine is lacking. The nucleus accumbens (NAc), a central hub of the mesolimbic reward circuitry, plays a pivotal role in mediating cocaine-related behaviors and synaptic plasticity. Moreover, due to their differences in expression pattern and downstream signaling pathways, dopamine receptor 1 (D1)- and D2-expressing medium spiny neurons (MSNs) play distinct roles in dopaminergic signal transduction and drug addiction (Kruyer et al., 2021; Tran et al., 2021). The heterogeneity of dopaminoceptive neurons and challenges in discovering signaling cascades present the major hurdles for identifying the precise neural mechanisms underlying ER stress-driven neurobehaviors in response to psychostimulants.

The activation of the UPR cascade triggers different cellular events through three classical UPR pathways: the inositol-requiring enzyme 1 (IRE1)/X-box binding protein 1 (XBP1), protein kinase RNA-like ER kinase (PERK)/eukaryotic translation initiation factor 2 alpha (eIF2α)/activating transcription factor 4 (ATF4) and ATF6 pathways (Shayya et al., 2022). Among the components of these pathways, ATF4 is a key downstream effector of ER stress and integrates stress responses (Demmings et al., 2021). In neurons, ATF4 can orchestrate the transcription of a plethora of target genes involved in memory formation and synaptic plasticity. Studies have suggested that the components of the UPR not only restore the ER proteostatic network but also crosstalk with intracellular lipid metabolism in neuronal cells (Celik et al., 2023).

Sphingolipids are pivotal constituents of synaptic structures, which play roles in synaptic dynamics and neuronal transmission (Wang W. et al., 2023). Most sphingolipids are synthesized *via* a *de novo* pathway in the ER, and serine palmitoyl transferase (SPT) functions as the initiating and rate-limiting enzyme (Hannun and Obeid, 2018; Tran et al., 2024). SPT catalyzes the condensation of serine and palmitoyl-CoA to form 3-ketosphinganine, which is further processed to produce ceremide and various sphingolipids. Studies have shown that addictive substances alter sphingolipid levels in certain brain regions of mice as well as individuals with substance use disorders (Kalinichenko et al., 2018). Eukaryotic SPTs consist of the serine palmitoyl transferase long-chain base subunit 1 (SPTLC1), SPTLC2 and SPTLC3 subunits. Of these, the SPTLC1 subunit is essential and associates with SPTLC2 or SPTLC3 to form active enzymes with different properties (Xie et al., 2023). As a key metabolic enzyme for sphingolipid biosynthesis within the ER, SPT activity has been shown to affect neuronal development and degeneration (Dohrn et al., 2024; Lone et al., 2022; Esch et al., 2023).

In this study, we identified the ER stress-related transcription factor ATF4 in D1-MSNs of the nucleus accumbens (NAc) plays a critical role in the development of cocaine induced neuroadaptation. ATF4 promotes the *de novo* sphingolipids synthesis by transcriptionally upregulating *Sptlc1*. Conversely, genetic knockdown of either *Sptlc1* or *Atf4* in D1-MSNs attenuates cocaine-induced behavioral and neuroplastic adaptations. These findings uncover a previously unappreciated ATF4–SPTLC1 signaling axis that links ER stress to sphingolipid metabolism, contributing to the maladaptive neuronal plasticity underlying cocaine-induced behaviors.

## 2 Materials and methods

### 2.1 Animals

For mouse experiments, male C57Bl6/J mice, or transgenic strains congenic with a C57 background, were used. Male C57BL/6J mice (6–8 weeks old) were purchased from Vital River Laboratory Animal Technology Co., Ltd. (Beijing, China). D1R-Cre mice (C57BL/6JGpt-Drd1-P2A-iCre, strain ID: T052793) and their wild-type littermates were obtained from GemPharmatech Co., Ltd. (Nanjing, China). All the mice were housed individually in a temperature- and humidity-controlled environment, with free access to water and food *ad libitum*. Experiments were conducted during the light phase of a 12 h light-dark cycle. All animal experiments were approved by the Institutional Animal Care and Use Committee (IACUC) of West China Hospital, Sichuan University, approval number 20230303060. All efforts were made to minimize the suffering of the animals.

### 2.2 Drugs

Cocaine-HCl was purchased from NIFDC (Beijing, China) and dissolved in 0.9% saline (2 mg/mL). The SPT antagonist, myriocin (#HY-N6798, Med Chem Express) was dissolved in saline containing 5% (V/V) DMSO to a final concentration of 10 mg/mL as a stock. 4-PBA (#HY-A0281, Med Chem Express) was used in a concentration of 9 mg/mL from the freshly prepared solution in 0.1% DMSO.

### 2.3 Locomotor activity

**FIGURE 1:**
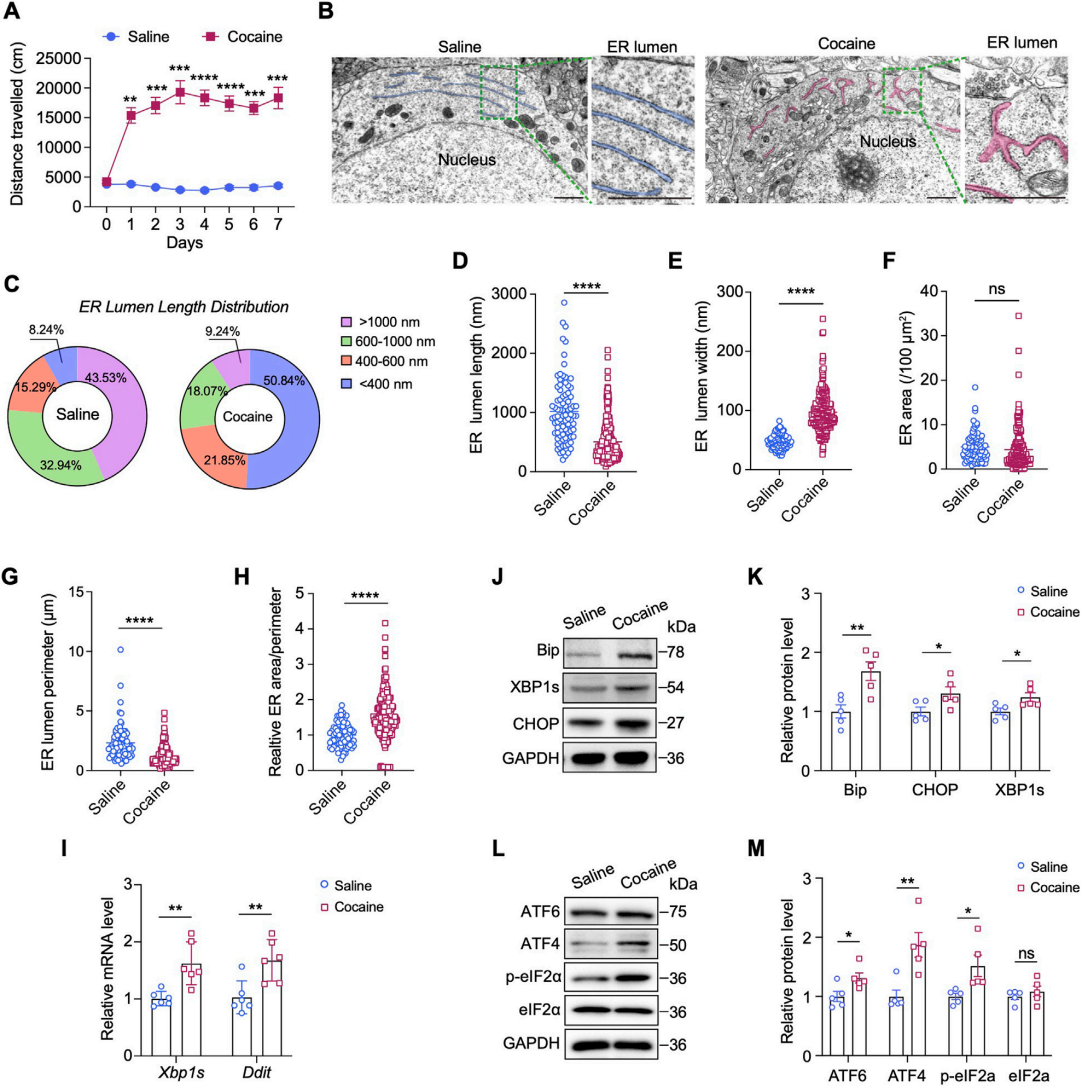
Cocaine induces ER stress in the NAc. **(A)** Locomotor activity in mice treated daily with cocaine (20 mg/kg). Data show mean distance traveled (cm) over 15 min (n = 10). Blue: saline-treated controls. **(B)** TEM images of the ER in NAc neurons. For each group: left, scale bar = 1 μm; right: enlarged images of the corresponding box region on the left, scale bar = 1 μm. ER: endoplasmic reticulum. The ER is highlighted and colored for better visualization. For each group, three mice were analyzed (n = 3), and a total of ten neurons per group were imaged and quantified. **(C)** Pie charts of the percentages of different length ranges of ER in **B**. **(D,E)** Quantification of the lengths **(D)** and widths **(E)** of ER: length, *t* (109.5) = 10.11, *p* < 0.0001; width, *t* (316) = 19.44, *p* < 0.0001. **(F–H)** Quantification of the areas **(F)**, perimeters **(G)** and the ratio of areas/perimeters **(H)** of ER after cocaine or saline treatment: area, *t* (197.1) = 1.344, *p* = 0.1805; perimeter, *t* (100.6) = 7.066, *p* < 0.0001; ratio of areas/perimeters, *t* (281.1) = 9.126, *p* < 0.0001. **(I)** mRNA levels of the *Xbp1*s and *Ddit* genes in the NAc: *Xbp1*s, *t* (10) = 3.438, *p* = 0.0063; *Ddit*, *t* (6.103) = 3.813, *p* = 0.0086. The results were normalized to GAPDH, and values are presented as fold change relative to the saline group (set to 1) (n = 6). **(J,K)** Western blot bands **(J)** and quantification **(K)** of ER stress-related proteins in the NAc. Quantitative analysis of Western blot bands was performed by normalizing target protein intensity to GAPDH, the mean value of the saline group was set to 1, and all other groups are expressed as fold change relative to saline: BiP, *t* (8) = 3.527, *p* = 0.0078; CHOP, *t* (8) = 2.343, *p* = 0.0472; XBP1s, *t* (8) = 2.573, *p* = 0.033 (n = 5). **(L,M)** Western blot bands **(L)** and quantification **(M)** of ATF6 and genes involved in eIF2α-ATF4 pathway in the NAc: ATF6, *t* (8) = 2.582, *p* = 0.0325; ATF4, *t* (8) = 3.729, *p* = 0.0058; p-eIF2α, *t* (4.696) = 2.742, *p* = 0.0435; eIF2α, *t* (8) = 0.8447, *p* = 0.4228 (n = 5). Data are presented as mean ± SEM. * *p* < 0.05; ** *p* < 0.01; *** *p* < 0.001; **** *p* < 0.0001 by unpaired two-tailed *t*-test (Welch’s correction when appropriate) vs. saline group; ns, *p* > 0.05.

Locomotor activity sessions were conducted once daily for seven consecutive days as previously described (Zhu et al., 2018). Briefly, each mouse was handled and placed in a black acrylic chamber (48 cm × 48 cm × 31 cm) and habituated to the environment for 2 days before testing. On test day, mice were received an intraperitoneal (i.p.) injection of saline or cocaine (20 mg/kg) and placed in the test chamber immediately, followed by locomotor activity was measured for 15 min automatically with a digital camera using EthoVision software (Noldus). To investigate the effect of myrioncin and 4-PBA on cocaine-induced locomotor activity, myriocin (0.1 or 0.3 mg/kg, i.p.) (Cinar et al., 2014; Dasgupta et al., 2020) or 4-PBA (90 mg/kg, i.p.) (Inceoglu et al., 2015; Wang Y. et al., 2018) was injected 30 min before each cocaine administration. Meanwhile, the control mice were injected with the same volume of solvent. Cocaine induced models were generated in multiple independent batches and allocated to different experimental paradigms. In some cases, tissues from the same animals were used for multiple assays, but statistical analyses were performed only once per animal. For example, Western blot analyses in Figure 1G,J,L were derived from the same cohort of mice, and tissue sections from the same cohort were also used for RNAscope SPTLC1 analysis (Figure 5C) and ATF4 nuclear localization (Figure 7D). These data were analyzed independently and are not double counted in any statistical comparisons.

### 2.4 Conditioned place preference (CPP)

Classical CPP experimental procedures were conducted in a three-chamber apparatus as previously described (Li et al., 2022). The apparatus consisted of two large compartments (16.8 cm × 12.7 cm × 12.7 cm) and one small compartment (7.2 cm × 12.7 cm × 12.7 cm). All animals were allowed to be habituated to the test room and handled for 2 days before any treatment. The time spent in each compartment was recorded to evaluate the initial unconditioned preference (day 0). Mice spending over 500 s in either compartment were excluded. The next 6 days (days 1–6) constituted the conditioning phase. In the CPP paradigm, saline was paired with the preferred compartment and cocaine was paired with the other compartment. Mice in the cocaine group received 20 mg/kg cocaine (i.p.) and saline injections alternately, and mice in the saline group received saline injections each day. Immediately after the injection, mice were restricted to the appropriate compartment for 30 min. On the test day (day 7), mice were allowed to freely explore the three compartments for 15 min without injections. The time spent in each compartment was recorded and the CPP score was calculated as the time spent in the cocaine-paired chamber during the post-test minus the time spent in this chamber during the pre-test. To measure the effect of myriocin or 4-PBA on cocaine CPP acquisition, myriocin (0.1 or 0.3 mg/kg, i.p.) or 4-PBA (90 mg/kg, i.p.) were injected 30 min before each CPP conditioning.

### 2.5 Sample collection

All mice were anesthetized with an intraperitoneal injection of sodium pentobarbital (75 mg/kg). For cocaine-induced locomotor activity experiments, mice were sacrificed within 2 h after the last cocaine injection. For CPP experiments, mice were sacrificed within 2 h after the CPP test on Day 7 (24 h after the last cocaine injection). For biochemical assays, the NAc was rapidly dissected using a 1.5 mm diameter brain punch under a stereomicroscope, immediately snap-frozen in liquid nitrogen, and stored at −80 °C until analysis. For histological and Golgi staining, as well as TEM, brains were rapidly collected and processed as described.

### 2.6 Sphingolipids extraction

Mice were sacrificed and the brains were rapidly removed. Sphingolipids were extracted from NAc tissue according to a previous report with modifications (Shaner et al., 2009). Briefly, NAc tissue (2.5 mg) was added with extraction solution [isopropanol: water: ethyl acetate = 3:1:6 (v/v/v)], and ultrasonicated in an ice-cold water bath. Samples were centrifuged at 6,000 g at 4 °C for 10 min, and the supernatants were transferred to a clean tube (Solvent I). The remaining lower layer was re-extracted with 2 mL of extraction solution [isopropanol: water: ethyl acetate = 3:1:6 (v/v/v)], and centrifugation at 6,000 g for 10 min. The supernatant was transferred to another new EP tube (Solvent II). The Solvent I and Solvent II were homogeneously transferred to the same EP tube and dried with a gentle stream of nitrogen gas.

### 2.7 LC-MS/MS assay

The dry residue was dissolved in 100 μL of 0.2% formic acid methanol solution containing 1 mM ammonium formate. Reconstituted extract (5 μL) was injected into the Waters ACQUITY UPLC system for analysis. The separation was performed on an Agilent Eclipse Plus C18 column (100 × 2.1 mm, 1.8 μm) with maintained at 55 °C by gradient elution with a mobile phase flow rate of 0.5 mL/min. Gradient elution mobile phases consisted of A (2 mM ammonium formate in water containing 0.2% formic acid) and B (1 mM ammonium formate in methanol containing 0.2% formic acid). A typical run time was 20 min. Mass spectrometric detection was accomplished both in positive ion mode and negative ion mode with ESI using Xevo G2-S Q-TOF (Waters Corp., Milford, United States). ESI conditions were optimized as follows: capillary voltage 3500 V, nozzle voltage 300 V, nebulizer pressure 40 psi, drying gas 50 L/h, and gas temperature 300 °C. The parameters were as follows: desolvation gas, 1000 L/h; desolvation temperature, 500 °C; cone gas, 150 L/h; source temperature, 150 °C; capillary voltage, 2.5 kV; cone voltage, 20 V for SM 16:0 (d18:1/16:0) and SM 18:0 (d18:1/18:0) and 30 V for SM 24:0 (d18:1/24:0). Data were acquired by Waters MassLynx (version 4.2). For multiple reaction monitoring, precursor-to-product ion transitions of m/z 703.42 → 184.01 for SM 16:0 (d18:1/16:0), of m/z 731.44 to 184.01 for SM18:0 (d18:1/18:0), of m/z 815.53 to 184.01 for SM 24:0 (d18:1/24:0), and of m/z 552.60→264.28 for ceramide C17 were used. Concentrations of the calibration standards, quality controls, and unknowns were evaluated by Waters MassLynx software (version 4.2). Data acquisition and processing were accomplished using Masslynx (Waters Corp., United States) (Liu et al., 2021; Lin et al., 2017).

### 2.8 Lipidomic data processing and analysis

Progenesis QI software (Waters) was used to analyze the lipidomic data acquired from UPLC/Q-TOF MS, and the lipids were identified from Lipid Maps Database (www.lipidmaps.org) and the Human Metabolome Database (http://www.hmdb.ca/) according to their MS characteristics. Datasheets from Progenesis QI software were obtained and absolute intensities of all identified compounds (normalized abundance) were recalculated to relative abundances of lipid molecules (vs. saline group). Pareto scaling was used for the final statistical models. The data were processed by supervised partial least-squares discriminate analysis methods to obtain group clusters. Lipid molecules with the highest impact on the group clustering were identified in the variable importance (VIP) plots (VIP > 1). An optimized false discovery rate approach was applied to control the false positive.

### 2.9 Virus and stereotaxic injection

The follow shRNAs were produced by Obio Technology (Shanghai, China): pAAV-CBG-DIO-EGFP-miR30shRNA targeting *Sptlc1* (shSptlc1, 2.04 × 10^12^ genomic copies/mL, sequence: 5′- GAG​CAC​TAT​GGG​ATC​AGT​ATT-3′); *Atf4* (shATF4, 2.04 × 10^12^ genomic copies/mL, sequence: 5′- CCA​GAG​CAT​TCC​TTT​AGT​TTA-3′); pAAV-CBG-DIO-EGFP-miR30shRNA (Scramble)-WPRE (shSc, 2.04 × 10^12^ genomic copies/mL). Briefly, mice were anesthetized with sodium pentobarbital (1%, w/v) and positioned in a stereotaxic apparatus. Under microscopic guidance, the scalp was incised and burr holes were drilled at the following coordinates targeting the NAc: anteroposterior (AP): +1.45 mm, mediolateral (ML): ±1.20 mm, dorsoventral (DV): −4.50 mm from bregma. A total of 300 nL of viral solution was bilaterally infused into the NAc over 10 min using a Hamilton syringe. The injection needle was retained in place for an additional 10 min to prevent reflux before slow withdrawal. All mice were allowed to recover from the surgery for at least 7 days and expression occurred in the injected brain region for approximately 4 weeks before experimentation.

### 2.10 Quantitative real time-PCR (qRT-PCR)

Total RNA was extracted from the brain tissue using 1 mL TRIzol reagent (Invitrogen) according to manufacturer’s instructions. Before proceeding with the RT-qPCR, RNA concentration was determined with the NanoDrop2000 UV-Vis Spectrophotometer (Thermo Fisher Scientific). cDNA was generated from 1 μg total RNA using Superscript III reverse transcriptase (Invitrogen). Quantitative real time-PCR (qRT-PCR) was performed using a SYBR-green PCR mix (Q711-2, Vazyme, Nanjing, China) with the system instrument (Thermo Fisher Scientific). The primer pairs were designed using the NCBI primer Blast algorithm. Primer pairs were synthesized by Tsingke Biotechnology (Beijing, China). Three technical replicates for each biological replicate were assessed. The expression level of each target gene was normalized to GAPDH and expressed as fold change relative to the saline group using the 2^−ΔΔCT^ method. The sequences of the qRT-PCR primers are shown in Supplementary Table S1.

### 2.11 Western blot

Proteins were extracted from NAc region using RIPA lysis buffer containing protease inhibitor, followed by centrifugation at 12,000 g for 20 min at 4 °C. The concentration of each sample was determined by a Pierce BCA Protein Assay Kit (Thermo Fisher Scientific). Equal amounts of protein were separated by 10% SDS-PAGE gel and transferred to PVDF membranes. After blocking in TBS containing 5% (wt/vol) nonfat dry milk for 1 h, membranes were incubated overnight at 4 °C with primary antibodies: Anti-BiP (1:1,000, Abclonal, #A0241), Anti-XBP1s (1:1,000, Abclonal, #A1731), Anti-CHOP (1:1,000, Abclonal, #A11346), Anti-ATF6 (1:1,000, Abclonal, #A0202), Anti-ATF4 (1:1,000, CST, #11815S), Anti-p-eIF2α (1:1,000, Abclonal, #AP0692), Anti-eIF2α (1:1,000, Abclonal, #A0764), Anti-SPTLC1 (1:1,000, Proteintech, #15376-1-AP), Anti-GAPDH (1:2000, CST, #2118), or Anti-Histone H3 (1:1,000, CST, #9715). Blots were incubated with HRP-conjugated secondary antibodies and visualized using enhanced chemiluminescence. Band intensities were quantified using ImageJ and normalized to GAPDH or Histone H3 and expressed as fold change relative to the control groups.

### 2.12 Luciferase reporter assay

Two *Sptlc1* promoter mutants were cloned upstream of a firefly luciferase reporter gene in the pGL3-control vector (Promega). The coding sequence of ATF4 was inserted into the pcDNA3.1 expression vector. 293T cells were seeded into 24-well plates and co-transfected with plasmids containing *Sptlc1* promoter mutants and either ATF4 overexpression plasmids or the empty control plasmids using the Lipofectamine 2000 (Invitrogen). After 48 h, cells were harvested, and the luciferase activities were determined by a dual-luciferase reporter assay kit (Promega, E1910) and Dual-Glo Luciferase system (Promega). Each group was conducted in triplicate. The firefly luciferase activity was normalized to Renilla luciferase for each well.

### 2.13 Chromatin immunoprecipitation (ChIP)

ChIP was conducted with the SimpleChIP Plus Enzymatic Chromatin IP Kit (#9005, CST) according to the manufacturer’s protocol. Briefly, brain samples were crosslinked with 1% formaldehyde and subject to 500 µL lysis buffer. The DNA and proteins were broken into fragments between 300 and 1,000 bp by ultrasonic shearing. The lysis was centrifuged at 10,000 g at 4 °C, and the supernatant was incubated with the anti-ATF4 antibody at 4 °C overnight. A normal rabbit IgG (CST, #2729) was added as a negative control antibody. After washing, the mixture was incubated with elution buffer at 62 °C for 2 h and then at 95 °C for 10 min. Then DNA was purified from the elution and was subject to RT-qPCR. The primers are listed in Supplementary Table S1. PCR was performed with equal amounts of specific antibody immunoprecipitated sample, control (IgG) and input. Values were normalized to input measurements and enrichment was calculated using the comparative 2^−ΔΔCT^ method.

### 2.14 SPT activity assay

SPT enzyme activity of NAc was quantitatively measured using the commercial ELISA kits in accordance with the manufacturer’s instructions (Best biological, Nanjing, China). Briefly, homogenates of the NAc tissue were prepared and centrifuged at 16,000 g for 5 min. The supernatants were collected and used for the detection of SPT activity. Briefly, supernatants were serially diluted in PBS in a microplate. After the plate was incubated at 37 °C for 1 h, 50 µL of assay buffer was added to each well. The reactions were developed at room temperature for 30 min under light-protected conditions. Stop solution was added to each well to terminate the reaction and the absorbance (OD) of each well at 450 nm was used to calculate the activity based on the standard curve. The standard curve was generated using SPT standard concentrations of 0.75, 15, 30, 60, and 120 U/mL. The protein content of each sample was detected by a BCA kit (Thermo Fisher Scientific). Data obtained from brain homogenates is expressed as U/mg.

### 2.15 Transmission electron microscopy (TEM)

Three mice in each group were applied for the observation of ER stress in the NAc by TEM as described previously (Wang L. et al., 2023). Briefly, mice brains containing NAc were harvested, immediately sliced into 1 mm^3^ slabs, and post-fixed in 2.5% glutaraldehyde overnight at 4 °C. Subsequently, the tissues were fixed in 1% osmium tetroxide solution, dehydrated in graded ethanol and flat-embedded in epon 812 (EMS Co., Ltd., Washington). Ultra-thin sections (50 nm) were made and double stained with uranyl acetate and lead citrate. Sections were then examined, and the typical images were taken under a Hitachi 7100 transmission electron microscope (Olympus, Tokyo, Japan).

### 2.16 Immunohistochemistry

Mice were anesthetized with pentobarbital sodium (i.p.) and transcardially perfused with ice-cold 0.9% saline, followed by 4% PFA. Extracted brains were fixed in 4% PFA overnight and then cryoprotected overnight in 30% sucrose in PBS. NAc region sections (20 μm-thick) were cut on a freezing microtome (Leica). Sections were incubated at 4 °C overnight with primary antibodies: Anti-SPTLC1 (1:100, Proteintech, #15376-1-AP), Anti-ATF4 (1:200, CST, #11815S), Anti-NeuN (1:400, Merck, #MAB377), Anti-D1R (1:200, Novus, #NB110-60017), Anti-D2R (1:200, Santa Cruz, # SC-5303). Sections were then incubated with the secondary antibodies (donkey anti-mouse IgG and donkey anti-rabbit IgG) diluted in PBS at room temperature for 1 h. Slices were counterstained with DAPI and coverslipped for fluorescent image acquisition. Images were captured using a Leica laser-scanning confocal microscope with a ×20/0.4 NA objective. Gain, threshold and black levels were not subjected to change during individual experiments. For SPTLC1 cellular expression analysis, images were analyzed using ImageJ (NIH, Bethesda, MD). The proportion of SPTLC1^+^ cells co-expressing D1R, D2R, or NeuN was calculated relative to the total number of SPTLC1^+^ cells within each section. Quantification was performed on four sections per mouse from three mice.

### 2.17 Golgi–cox staining

Golgi–cox staining was performed using the FD Rapid Golgi StainTM kit (FD Neuro Technologies, United States) according to manufacturer’s instructions. Brains were immersed in impregnation solution for 2 weeks at room temperature in the dark, followed by incubation in Solution C for 72 h. Coronal sections (100 μm) were cut using a freezing microtome (Leica CM1950) and mounted on gelatin-coated slides. Sections were subsequently stained with Solutions D and E. MSNs in the NAc core region were identified based on morphology (large round/oval soma with branched dendrites and abundant spines) and GFP expression. Neurons from 5 to 8 rostrocaudally matched sections per mouse were imaged. Morphometric analysis was performed in ImageJ using the SNT plugin. Dendritic length, spine density, and the ratio of dendritic endpoints to total dendrite length were quantified from skeletonized images. Sholl analysis was conducted with a starting radius of 10 μm, ending radius of 140 μm, and 10 μm intervals. A total of 15 neurons per group were analyzed.

### 2.18 RNAscope *in situ* hybridization (ISH)

RNAscope ISH was conducted to label D1 and D2 MSN neurons in the NAc. Frozen tissue sections (20 μm thick) prepared for immunohistochemistry were used for RNAscope ISH. *Drd1* and *Drd2* mRNAs were detected using specific RNAscope probes (ACD Bio; #461908 and #406509, respectively). For SPTLC1 or ATF4 co-staining, sections were pre-treated according to the protocol, and subsequently incubated with primary antibodies overnight: Anti-ATF4 (1:100, CST, #11815S), Anti-SPTLC1 (1:100, Proteintech, #15376-1-AP), followed by *Drd1* and *Drd2* mRNA staining. The *Drd1* mRNA was labeled with Opal 690 and the *Drd2* mRNA with Opal 570. Protein signals were detected using Alexa Fluor 488-conjugated secondary antibodies (1:1,000, Thermo Fisher Scientific). ProLong Gold Anti-Fade Reagent mountant (Invitrogen) was used to preserve signals. Images were taken at least three equal-sized squares using a Leica confocal microscope at ×20 magnification. Quantification of ATF4 signals was performed within *Drd1* ^+^ or *Drd2* ^+^ neuronal populations. Specifically, nuclear ATF4 fluorescence intensity (defined within DAPI-stained nuclei) was measured in *Drd1* ^+^ or *Drd2* ^+^ cells using ImageJ. Analysis was performed on six mice, with approximately 150 cells quantified per mouse.

### 2.19 Statistical analysis

All data are presented as means ± SEM. All statistical analyses were carried out using GraphPad Prism 9. For all experiments, ANOVAs were performed. To evaluate significance of two groups, an unpaired *t*-test was used, with Welch’s correction applied when necessary. For multiple comparisons between groups, we used one-way ANOVA followed by an appropriate *post hoc* test. For repeated multiple comparisons data were analyzed using a two-way ANOVA (with repeated measures where appropriate) followed by an appropriate *post hoc* test. The null hypothesis was rejected at *p* < 0.05 and indicated by asterisks in the figures.

## 3 Results

### 3.1 Cocaine induces neuronal ER stress in the NAc of mice

We established a typical model of cocaine-induced hyperlocomotor activity by administering cocaine (20 mg/kg, i.p.) to mice daily for seven consecutive days. Locomotor activity was quantified during the first 15 min after each injection and we observed robust locomotor activation in cocaine-treated mice compared with saline controls (Figure 1A). As structural and morphological changes in the ER have important functional implications for ER stress, we first applied TEM to evaluate morphological changes of the ER in the NAc after repeated cocaine injections. Brain tissues were collected within 2 h after the last injection for ultrastructural analysis. The saline-treated mice exhibited a typical, well-developed, and rough ER morphology in the neurons. In contrast, the cocaine-treated mice exhibited an obviously altered ER morphology, characterized by fragmentation, dilatation and thickened and swollen cisternae (Figure 1B). Statistical analysis revealed that cocaine reduced the length of the ER lumen, resulting in short ER fragments with a maximum length less than 200 nm (*p* < 0.0001) (Figures 1C,D). We then analyzed ER dilatation by measuring the width of the largest lumen in each neuronal cell body. Compared with that in the saline group (47.97 ± 1.31 nm), the width of the ER lumen was significantly increased in neurons of cocaine-treated mice (96.93 ± 2.14 nm, *p* < 0.0001) (Figure 1E). Moreover, the perimeter of the ER was notably decreased by cocaine (*p* < 0.0001), while the cavity area remained unchanged (*p* = 0.18) (Figures 1F,G). Additionally, the ratio of area to perimeter was significantly altered, suggesting increased fragmentation or disruption of ER morphology (*p* < 0.0001) (Figure 1H). Overall, these ultrastructural observations suggest that neuronal cells undergo ER stress in response to cocaine.

In response to ER stress, membrane-bound sensor proteins, such as IRE1, PERK, ATF4 and ATF6, are activated to initiate the UPR signaling cascade (Jiang et al., 2015; Shen et al., 2002). To identify the sensor proteins activated by cocaine, we examined the expression of ER stress-related genes and the potential downstream UPR pathway. Compared with saline, cocaine significantly increased the transcript levels of spliced *Xbp1* (*Xbp1s, p* < 0.01) and DNA damage-inducible transcript 3 (*Ddit, p* < 0.01), two classical ER stress-induced genes, in the NAc (Figure 1I). Consistently, the protein levels of CHOP (encoded by *Ddit*) and XBP1s, as well as those of binding immunoglobulin protein (BiP), an ER chaperone, were also elevated (*p* < 0.05) (Figures 1J,K). Furthermore, the protein levels of ATF6 and ATF4 were obviously increased in the NAc of mice after cocaine administration, and the level of phosphorylated elF2α (p-elF2α) was also apparently increased (*p* < 0.05) (Figures 1L,M). Notably, the expression of ATF4 (*p* < 0.01) was approximately 2-fold greater than that of ATF6, indicating a greater activation of ATF4 in response to cocaine. Taken together, these findings show that cocaine significantly promotes ER stress and elF2α–ATF4 pathway in the NAc.

### 3.2 Cocaine remodels sphingolipid metabolism and enhances SPTLC1 expression in the NAc

**FIGURE 2:**
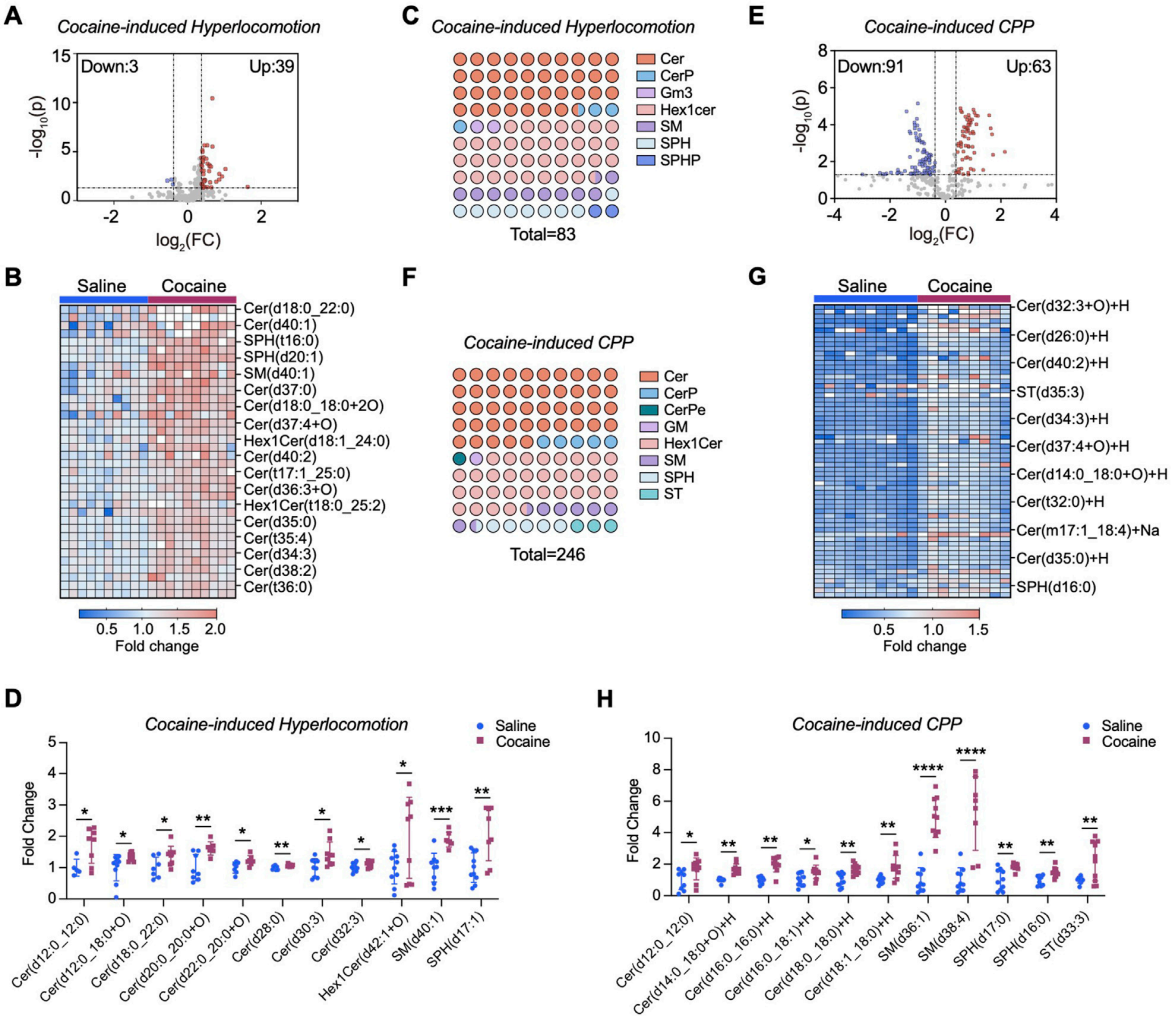
Cocaine reprograms sphingolipids metabolism in the NAc. **(A)** Volcano plot of sphingolipidomic profiling in the NAc of mice subjected to cocaine-induced hyperlocomotion. Lipid levels were normalized to the mean of the saline control group (set as 1) to provide a baseline for comparison. The *x*-axis represents the log_2_ fold change (FC) between the two groups, and the *y*-axis represents the −log_10_ of the *p*-value. Significant changes are defined by a threshold of |FC| > 1.3 and *p*-value <0.05 (calculated by unpaired two-tailed *t*-test). Upregulated lipids are shown in red, while downregulated lipids are shown in blue. Each point represents a lipid species (n = 10). **(B)** Heatmap displaying changes in upregulated sphingolipid species in the NAc of cocaine-induced hyperlocomotion. **(C)** Dot plot illustrating the altered sphingolipid subclasses in the brain of mice subjected to cocaine-induced hyperlocomotion. For each color, the number of dots reflects the relative proportion of that subclass among all significantly altered sphingolipids, while the total number of dots corresponds to the total abundance of altered lipid species. **(D)** Bar graph shows the intensity of a series of altered sphingolipids in the NAc of mice subjected to cocaine-induced hyperlocomotion. Values are normalized to the mean of the saline group, which was set to 1 (fold change relative to saline). **(E)** Volcano plot of sphingolipidomic analysis in the NAc of cocaine CPP mice (n = 10 for saline group, n = 9 for cocaine group). **(F)** Same as **(C)**, but in the NAc of mice subjected to cocaine-induced CPP. **(G)** Heatmap displaying changes in upregulated sphingolipid species in the NAc of cocaine CPP mice. **(H)** Bar graph shows the intensity of a series of altered sphingolipids in the NAc of cocaine CPP mice. Values are normalized to the mean of the saline group, which was set to 1 (fold change relative to saline). Data are presented as mean ± SEM. * *p* < 0.05; ** *p* < 0.01; *** *p* < 0.001; *** *p* < 0.0001 by unpaired two-tailed *t*-test vs. saline group.

Previous studies indicate ER stress modulates sphingolipid biosynthesis (Kim et al., 2022). To determine the link between ER stress and sphingolipid metabolism upon cocaine exposure, by using a LC‒MS/MS-based lipidomic approach, we measured the levels of ceramides and sphingolipid intermediates in the NAc. In the hyperlocomotor activity model (referred to cocaine-induced hyperlocomotion), alterations in 42 sphingolipid molecules were observed in the NAc of mice following repeated cocaine treatment (Figure 2A). Of these, 39 showed significantly increased levels, including species of ceramides, sphingosine (SPH), and sphingomyelin (SM) (Figures 2B,C; Supplementary Figure S1A). Ceramide levels showed the most prominent rise; for example, ceramide (d12:0–12:0) increased over 2-fold, and ceramide (d30:3) by 1.5-fold (Figure 2D). Levels of SM (d40:1) and SPH (d17:1) were also significantly elevated (*p* < 0.05). The mechanistic diagram of the sphingolipid metabolic pathway was shown in Supplementary Figure S2A, indicating that repeated cocaine exposure enhances *de novo* sphingolipid biosynthesis in the NAc.

We further validated these findings using the cocaine conditioned place preference (CPP) paradigm. NAc tissues were collected 24 h after the last injection, rapidly dissected, and prepared for LC–MS/MS profiling. LC‒MS/MS analysis revealed 154 sphingolipid molecules modified by cocaine (Figure 2E), among which a certain number of ceramide species, including ceramide (d12:0–12:0, d16:0–16:0, and d18:0–18:0), showed significantly increased levels (Figures 2F,G; Supplementary Figure S1B). In particular, the level of SM (d36:1) was markedly increased more than 4.5-fold (*p* < 0.0001), and that of sulfatide [ST (d33:3)] increased 2-fold (*p* < 0.01) (Figure 2H). Notably, the magnitude of sphingolipid alterations was more pronounced under the CPP paradigm compared with the hyperlocomotion paradigm, suggesting that distinct behavioral contexts may differentially engage lipid metabolic pathways in the NAc.

**FIGURE 3:**
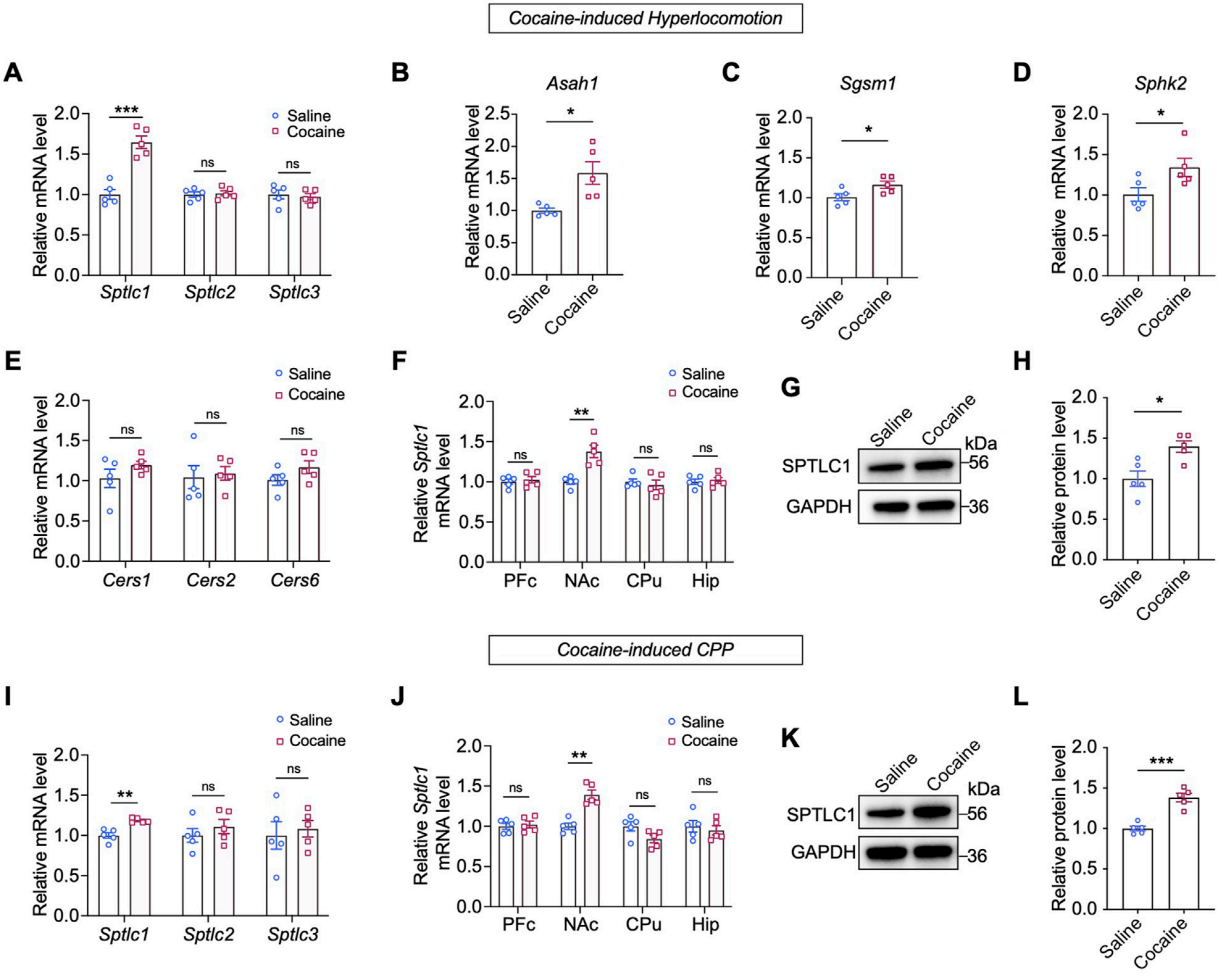
Cocaine upregulates the expression of SPTLC1 in the NAc. **(A)** Expression of *Sptlc1, Sptlc2* and *Sptlc3* genes in the NAc of mice subjected to cocaine-induced hyperlocomotion: *Sptlc1*, *t* (8) = 6.592, *p* = 0.0002; *Sptlc2*, *t* (8) = 0.3452, *p* = 0.7389; *Sptlc3*, *t* (8) = 0.416, *p* = 0.6915 (n = 5). **(B–E)** Expression of sphingolipid metabolic enzyme genes measured by qPCR: *Asah1*, *t* (4.397) = 3.243, *p* = 0.0275; *Sgsm1*, *t* (8) = 2.471, *p* = 0.0386; *Sphk2*, *t* (8) = 2.397, *p* = 0.0434; *Cers1*, *t* (8) = 1.342, *p* = 0.2163; *Cers2*, *t* (8) = 0.2793, *p* = 0.7871; *Cers6 t* (8) = 1.569, *p* = 0.1553 (n = 5). **(F)** Expression of *Sptlc1* genes measured by qPCR in four brain regions of mice: PFc, *t* (8) = 0.4973, *p* = 0.6342; NAc, *t* (8) = 4.612, *p* = 0.0017; CPu, *t* (8) = 0.5461, *p* = 0.5999; Hip, *t* (8) = 0.3283, *p* = 0.7511 (n = 5). **(G,H)** Western blot bands and quantification of the protein levels of SPTLC1 in the NAc: *t* (8) = 3.346, *p* = 0.0101 (n = 5). **(I)** Expression of *Sptlc1, Sptlc2* and *Sptlc3* genes in the NAc of cocaine CPP mice as measured by qPCR: *Sptlc1*, *t* (8) = 4.968, *p* = 0.0011; *Sptlc2*, *t* (8) = 0.9072, *p* = 0.3908; *Sptlc3*, *t* (8) = 0.4198, *p* = 0.6857 (n = 5). **(J)** Expression of *Sptlc1* levels measured by qPCR in four brain regions of cocaine CPP mice: PFc, *t* (8) = 0.4071, *p* = 0.6946; NAc, *t* (8) = 5.659, *p* = 0.0005; CPu, *t* (8) = 2.133, *p* = 0.0655; Hip, *t* (8) = 0.5418, *p* = 0.6027 (n = 5). **(K,L)** Western blot bands and quantification of SPTLC1 in the NAc of cocaine CPP mice: *t* (8) = 6.367, *p* = 0.0002 (n = 5). Abbreviations: PFc, prefrontal cortex; NAc, nucleus accumbens; CPu, caudate nucleus; Hip, hippocampus. Data are presented as mean ± SEM. All qPCR values were normalized to GAPDH, and Western blot signals were normalized to GAPDH. For both qPCR and Western blot, the saline group was set to 1 and all other groups are expressed as fold change relative to saline. * *p* < 0.05; ** *p* < 0.01; *** *p* < 0.001 by unpaired two-tailed *t*-test vs. saline group; ns, *p* > 0.05.

To determine if enzyme activity changes caused these sphingolipid alterations, we measured mRNA levels of related enzymes. As SPT, the rate-limiting enzyme for sphingolipid *de novo* synthesis, consists of SPTLC1, SPTLC2, and SPTLC3 subunits. We focused on their genes (Johnson et al., 2021). Interestingly, we observed a significant increase in the mRNA level of *Sptlc1* (*p* < 0.001) in the NAc after repeated cocaine injection, whereas the mRNA levels of *Sptlc2* and *Sptlc3* remained unchanged (*p* > 0.05) (Figure 3A). Other enzyme-encoding genes, such as N-acylsphingosine amidohydrolase 1 (*Asah1*), sphingomyelin synthase 1 (*Sgms1*) and sphingosine kinase 2 (*Sphk2*) also showed increased mRNA levels (*p* < 0.05) (Figures 3B–D), but ceramide synthases (*Cers1*, *Cers2*, and *Cers6*) were unaffected (Figure 3E). Notably, cocaine only increased *Sptlc1* mRNA in the NAc, not in the liver or other brain regions (Figure 3F; Supplementary Figure S2B). Consistently, SPTLC1 protein levels were significantly increased in the NAc following repeated cocaine administration (*p* < 0.05) (Figures 3G,H). Time-course analysis revealed a progressive increase in SPTLC1 after daily cocaine injections, peaking on Day 7 and returning to baseline by Day 14 of withdrawal (Supplementary Figure S2C–E). These findings showed that the dynamic regulation of SPTLC1 expression in the NAc may function as an adaptive response over the course of cocaine exposure and withdrawal.

We further confirmed *Sptlc1* upregulation in cocaine-conditioned mice using a CPP paradigm (Supplementary Figure S2F), with no change in *Sptlc2/3* expression (Figure 3I). Again, this effect was restricted to the NAc and absent in other brain regions or the liver (Figure 3J; Supplementary Figure S2G). Consistently, Western blot analysis also revealed that cocaine exposure increased SPTLC1 protein levels by approximately 1.5-fold in the CPP model (normalized to the saline vehicle group set as 1.0). These findings demonstrate that repeated cocaine exposure specifically upregulates SPTLC1 in the NAc.

### 3.3 Inhibition of SPT prevents cocaine-induced neurobehaviors and cocaine-reprogrammed sphingolipid metabolism

**FIGURE 4:**
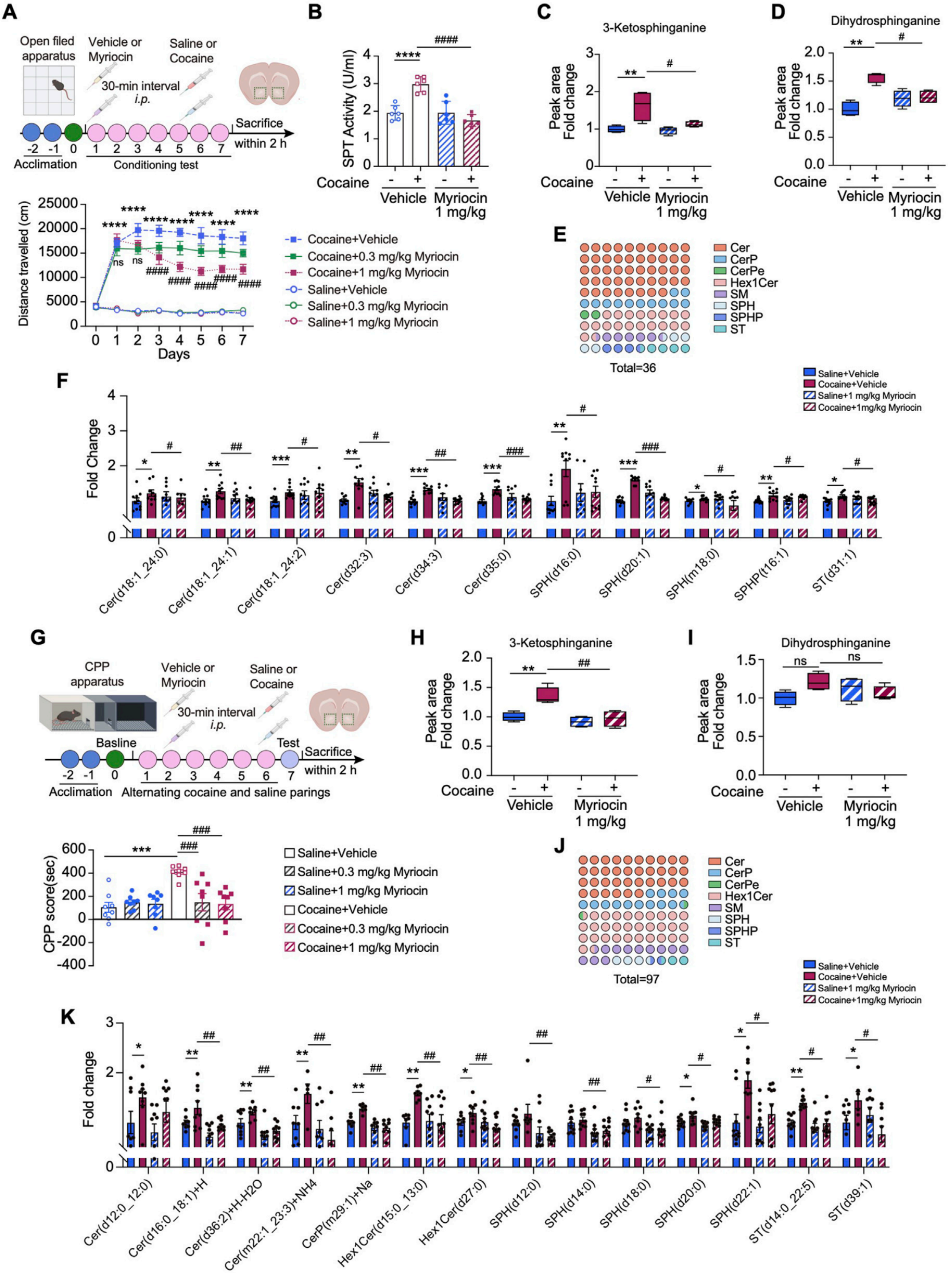
SPT inhibition represses cocaine-induced neurobehaviors and sphingolipid metabolism reprogramming in the NAc. **(A)** Upper: diagram of establishing cocaine-induced hyperlocomotor activity in mice with myriocin pretreatment. Lower: total distances of mice travelled before or after *i.p.* injection of 20 mg/kg cocaine with myriocin pretreatment (0.3, 1 mg/kg) and the vehicle control (n = 10). Mice received vehicle or myriocin 30 min before daily behavioral testing. **(B)** Bar graph shows the changes of SPT activity measured by ELISA assay in mice pretreated with myriocin (1 mg/kg) (n = 6). Main effects: cocaine, *F* (1,20) = 9.455, p = 0.006; myriocin, *F* (1,20) = 28.21, *p* < 0.0001; interaction, *F* (1,20) = 28.58, *p* < 0.0001. **(C,D)** Bar graph shows the levels of 3-ketosphinganine **(C)** and dihydrosphinganine **(D)** in the NAc of cocaine-treated mice with myriocin (1 mg/kg) pretreatment (n = 4). 3-ketosphinganine, main effects: cocaine, *F* (1,12) = 9.455, *p* = 0.006; myriocin, *F* (1,12) = 28.21, *p* < 0.0001; interaction, *F* (1,12) = 28.58, *p* < 0.0001. Dihydrosphinganine, main effects: cocaine, *F* (1,12) = 12.73, *p* = 0.0039; myriocin, *F* (1,12) = 5.8529, *p* = 0.0327; interaction, *F* (1,12) = 4.175, *p* = 0.0636. **(E)** The dot matrix chart shows eight minor categories of sphingolipids significantly changed by myriocin (1 mg/kg) pretreatment in cocaine-treated group. The color of the dots represents a lipid subclass and the quantity of the dots represents the percentage. **(F)** Bar graph shows the intensity of a series of changed sphingolipids in the NAc of mice with cocaine-induced hyperlocomotion (n = 10). **(G)** Diagram of establishing cocaine CPP model. Bar graph shows the cocaine CPP scores with myriocin pretreatment and the vehicle control (0.3, 1 mg/kg) (n = 8). **(H,I)** Bar graph shows the levels of 3-ketosphinganine **(H)** and dihydrosphinganine **(I)** in the NAc of cocaine CPP mice with myriocin (1 mg/kg) pretreatment (n = 4). 3-ketosphinganine, main effects: cocaine, *F* (1,12) = 11.57, *p* = 0.0052; myriocin, *F* (1,12) = 15.43, *p* = 0.0020; interaction, *F* (1,12) = 6.280, *p* = 0.0276; Dihydrosphinganine, main effects: cocaine, *F* (1,12) = 1.456, *p* = 0.2508; myriocin, *F* (1,12) = 0.0949, *p* = 0.7633; interaction, *F* (1,12) = 5.285, *p* = 0.0403. **(J)** The dot matrix chart shows eight minor categories of sphingolipids significantly modified by myriocin (1 mg/kg) of cocaine-treated mice. **(K)** Bar graph shows the intensity of a series of changed sphingolipids in the NAc of cocaine CPP mice with myriocin (1 mg/kg) pretreatment (n = 10). Data are presented as mean ± SEM and analyzed using two-way ANOVA followed by Turkey’s multiple comparisons test. * *p* < 0.05; ** *p* < 0.01; *** *p* < 0.01; **** *p* < 0.0001 vs. saline group. #*p* < 0.05. ##*p* < 0.01; ###*p* < 0.001 vs. cocaine group; ns, *p* > 0.05.

Given the upregulation of SPTLC1 and enhanced *de novo* synthesis of sphingolipids in the NAc upon cocaine exposure, we speculated that SPT may function in this process. To test this, we applied myriocin, the most potent inhibitor of SPT (Wadsworth et al., 2013), to inhibit SPT activity and then investigated its effects on cocaine-induced neurobehaviors. In the hyperlocomotor activity model, mice received i.p. myriocin (0.3 or 1 mg/kg) or vehicles 30 min before each cocaine or saline injection (Figure 4A). Myriocin (1 mg/kg) effectively attenuated cocaine-induced hyperlocomotion activity without affecting baseline activity, while the 0.3 mg/kg dose showed a decreasing trend (Figure 4A). Based on these results, we selected the 1 mg/kg dose of myriocin for subsequent analyses. ELISA revealed that cocaine increased SPT activity in the NAc, which was inhibited by myriocin (Figure 4B). SPT catalyzes the initial step in sphingolipid biosynthesis, forming 3-ketodihydrosphingosin from L-serine and palmitoyl-CoA (Johnson et al., 2021). LC–MS/MS showed that cocaine elevated levels of 3-ketodihydrosphingosin and dihydrosphingosine, and myriocin (1 mg/kg) treatment suppressed these increases (Figures 4C,D).

We further explored the role of SPT in cocaine-induced sphingolipid remodeling by analyzing the sphingolipid profile in the NAc using LC–MS/MS. Myriocin (1 mg/kg) significantly altered the levels of 36 sphingolipid species affected by cocaine, including Cer, SPH, SM, which are key hubs in sphingolipid metabolism (Figure 4E). Specifically, cocaine increased the levels of ceramide (d18:1–24:0, d18:1–24:1, d18:1–24:2, d34:3) and SPH (d20:1) (Figure 4F). Myriocin (1 mg/kg, i.p.) effectively reduced these elevated ceramides, without affecting basal sphingolipid levels in saline-treated mice (Figure 4F).

We next asked whether myriocin has a similar effect on repressing cocaine induced CPP. To this end, mice were injected (i.p.) with myriocin 30 min before each cocaine injection. Importantly, pretreatment with myriocin (0.3, 1 mg/kg) significantly attenuated cocaine CPP scores in mice (Figure 4G). Notably, we observed that CPP scores in saline-treated groups were slightly positive, suggesting a modest vehicle effect. Nevertheless, the CPP response induced by cocaine was markedly higher than the vehicle effect, indicating that the primary behavioral change is driven by cocaine. We also examined downstream SPT metabolites in the NAc. LC–MS/MS analysis showed that cocaine CPP increased 3-ketodihydrosphingosin levels, which were significantly reduced by myriocin (1 mg/kg, i.p.) (Figure 4H). Dihydrosphingosine levels were also slightly decreased by myriocin (1 mg/kg), though without statistical significance (Figure 4I). Myriocin (1 mg/kg) also altered 97 sphingolipid molecules in cocaine-treated mice, decreasing the levels of ceramide (d12:0/12:0) and others promoted by cocaine (Figures 4J,K). Collectively, these data indicate that reducing the *de novo* production of sphingolipids due to the inhibition of SPT activity can attenuate the effects of cocaine.

### 3.4 SPTLC1 is enriched in D1-MSNs, and its knockdown alleviates cocaine-induced neurobehaviors and dendritic alternations

**FIGURE 5:**
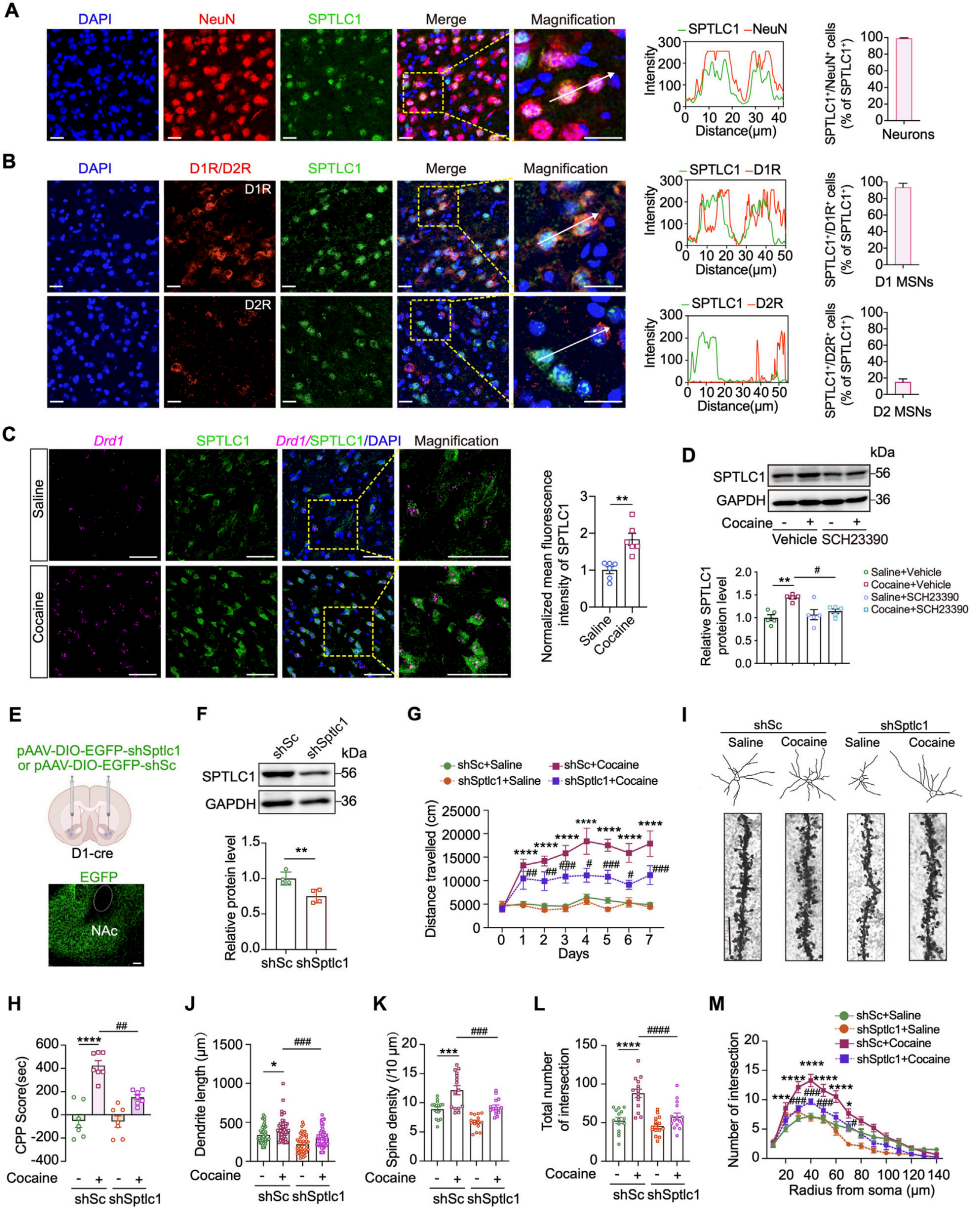
*Sptlc1* knockdown in the D1-MSN represses cocaine-induced neurobehaviors and dendritic remodeling. **(A)** Left: representative images showing the co-expression of SPTLC1 and NeuN in the NAc of naïve C57BL/6 mice. Scale bars: 20 μm. Middle: Co-localization analysis, presented as the distance distributions of fluorescence intensity. Right: Bar graph showing the percentage of SPTLC1-positive cells that are co-positive for NeuN (SPTLC1^+^NeuN^+^ cells/total SPTLC1^+^ cells) in the NAc (n = 3). **(B)** Left: representative images of co-expression of SPTLC1 and D1R or D2R in the NAc core region of naïve C57BL/6 mice. Scale bars: 40 μm. Middle: Co-localization analysis. Right: Bar graph showing the percentage of SPTLC1-positive cells that are co-positive for D1R (SPTLC1^+^D1R^+^ cells/total SPTLC1^+^ cells) and D2R (SPTLC1^+^D2R^+^ cells/total SPTLC1^+^ cells) in the NAc (n = 3). **(C)** Representative photographs of RNAscope *in situ* hybridization assays of *Drd1* mRNA and SPTLC1 protein in the NAc of mice subjected to cocaine-induced hyperlocomotion. Right: quantitative analysis of SPTLC1 fluorescence intensity: *t* (10) = 4.365, *p* = 0.0014 (n = 6). Scale bars: 30 μm. **(D)** Representative Western blot bands showing SPTLC1 and GAPDH (loading control) expression in the NAc of mice subjected to cocaine-indued hyperlocomotion after pretreatment with D1R inhibitor SCH23390 (administered 30 min prior to testing; n = 5). For quantitative analysis of SPTLC1 protein expression, band densities were normalized to GAPDH, and then further normalized to the saline vehicle group (set as 1.0) to represent relative SPTLC1 expression levels. Main effects: cocaine, *F* (1,16) = 13.43, *p* = 0.0021; SCH23390, *F* (1,16) = 2.730, *p* = 0.1180; interaction, *F* (1,16) = 6.925, *p* = 0.0181. **(E)** Upper: schematic diagram of the location of the injected virus. Lower: representative images for the AAV vector-mediated overexpression of EGFP in the NAc. Scale bars: 100 μm. **(F)** Representative Western blot bands and quantitative data analyses of SPTLC1 in the NAc after virus administration: *t* (6) = 3.776, *p* = 0.0092. (n = 4). **(G)** Average distance traveled within 15 min by shSc- and shSptlc1-treated D1-Cre mice subjected to cocaine-induced hyperlocomotion (n = 7). **(H)** CPP score of shSc- and shSptlc1-treated D1-Cre mice subjected to cocaine CPP paradigm (n = 7). Main effects: cocaine, *F* (1,24) = 61.65, *p* < 0.0001; shSptlc1, *F* (1,24) = 10.06, *p* = 0.0041; interaction, *F* (1,24) = 9.389, *p* = 0.0053. **(I)** Representative traces of Golgi–Cox-stained MSN neurons from the NAc core region of cocaine CPP mice. Enlarged areas showing the dendritic spine distribution and morphology of Golgi-stained MSNs. **(J–M)** The dendritic complicity of reconstructed MSNs using Sholl analysis (n = 15 neurons). Dendrite length, main effects: cocaine, *F* (1,176) = 16.76, *p* < 0.0001; shSptlc1, *F* (1,176) = 36.17, *p* < 0.0001; interaction, *F* (1,176) = 0.0885, *p* = 0.7664. Spine density, main effects: cocaine, *F* (1,56) = 34.35, *p* < 0.0001; shSptlc1, *F* (1,56) = 26.47, *p* < 0.0001; interaction, *F* (1,56) = 0.4294, *p* = 0.5150. Intersection number, main effects: cocaine, *F* (1,56) = 31.58 *p* < 0.0001; shSptlc1, *F* (1,56) = 19.17, *p* < 0.0001; interaction, *F* (1,56) = 7.439, *p* = 0.0085. Scale bars: 10 μm. Data are presented as mean ± SEM and analyzed using two-way ANOVA followed by Turkey’s multiple comparisons test. For **(C,F)**, data were analyzed using unpaired two-tailed *t*-test. *p < 0.05; **p < 0.01; ***p < 0.001; ****p < 0.0001 vs. saline group. #*p* < 0.05; ##*p* < 0.01 vs. cocaine group. ns, *p* > 0.05.

Since cocaine only increased SPTLC1 expression among SPT subunits in the NAc, we hypothesized that SPTLC1 may be pivotal in remodeling sphingolipid metabolism in response to cocaine. We first determined the location of SPTLC1 expression in the NAc of naïve mice. Immunostaining analysis showed that SPTLC1 was predominantly found in NeuN-positive neurons of the NAc, although not exclusively limited to them (Figure 5A). As approximately 95% of NAc neurons are GABAergic MSNs, which include D1 and D2 subtypes with distinct functions (Gangarossa et al., 2013), we further assessed SPTLC1 expression in these neuronal populations. Co-immunofluorescence with D1R or D2R antibodies was performed, and the proportion of SPTLC1-positive cells overlapping with D1R or D2R was quantified. These analyses indicated that SPTLC1 is more abundantly expressed in D1-MSNs (98%), whereas its expression in D2-MSNs is comparatively lower within the NAc core region (Figure 5B). Due to inherent limitations associated with D1R and D2R antibody labeling, we further validated these observations using RNAscope *in situ* hybridization with a *Drd1*-specific probe, combined with immunofluorescence staining of SPTLC1, quantification revealed that repeated cocaine administration strongly increased SPTLC1 expression in the NAc (Figure 5C).

To further explore the association between D1-MSNs and SPTLC1, we treated the mice with SCH23390, a D1R antagonist that has been proven to reduce cocaine-induced hyperlocomotion and CPP. Interestingly, treatment with the D1R antagonist SCH23390 (0.01 mg/kg, i.p.) prior to cocaine injection attenuated cocaine induced SPTLC1 upregulation in the NAc (*p* < 0.05). (Figure 4D), indicating crosstalk between SPTLC1 and D1R pathways.

The observation that SPTLC1 is more abundantly expressed in D1-than in D2-MSNs in the NAc motivated us to investigate whether SPTLC1 contributes to the cocaine effect in a D1-MSN-dependent manner. To this end, we applied an AAV-mediated cell type-specific gene knockdown strategy that allowed the overexpression of shRNA targeting *Sptlc1* (AAV-shSptlc1) in D1-Cre mice (Figure 5E). We bilaterally injected AAV-DIO shSptlc1 EGFP (referred to shSptlc1) or AAV-DIO shScramble EGFP (control, referred to shSc) into the NAc of transgenic mice in which Cre expression is driven by the *Drd1*, and then measured EGFP expression 3 weeks after AAV infection. As expected, DIO-EGFP expression was restricted to the NAc (Figure 5E; Supplementary Figure S3A). Furthermore, co-immunofluorescence with a D1R antibody showed that EGFP signal predominantly colocalized with D1-MSNs, confirming neuronal subtype-specific expression (Supplementary Figure S3B). Immunoblotting revealed that AAV-shSptlc1 significantly reduced the SPTLC1 protein level in the NAc (*p* < 0.01) (Figure 5F). To characterize the functional role of SPTLC1 during locomotion, D1 MSN-specific SPTLC1-knockdown mice were subjected to repeated cocaine injections (i.p.), followed by locomotor activity test. Importantly, knockdown of *Sptlc1* in D1-MSNs effectively suppressed not only cocaine-induced hyperlocomotion activity (Figure 5G) but also the rewarding effect of cocaine (*p* < 0.01) (Figure 5H). These data reveal a critical role of SPTLC1 in D1-MSNs in the cocaine-induced behavioral responses.

Dendritic structural changes in the NAc are necessary for the long-lasting behavioral adaptations induced by drugs (Cahill et al., 2018). We next investigated the effect of *Sptlc1* knockdown on dendritic remodeling in cocaine addiction. Three weeks after AAV-shSptlc1 (or AAV-shSc) virus injection, the mice were subjected to repeated cocaine injections. Mouse brains were isolated at the end of the test and subjected to Golgi-Cox staining Considering the limitations of Golgi-Cox staining for discriminating neuron subtypes, we selected MSNs expressing EGFP for the following analysis. Notably, compared with saline treatment, cocaine significantly increased dendritic complexity in these MSNs, as indicated by increases in both dendritic length and spine density (*p* < 0.05) (Figures 5I–L); in addiction, cocaine also induced dendritic arborization (Figure 5M), these effects were significantly reversed by *Sptlc1* knockdown. Collectively, our findings show that SPTLC1 functions as a key regulator of sphingolipid metabolism and dendritic plasticity which are essential for the development of cocaine behaviors.

### 3.5 ER stress‒activated ATF4 enhances the transcription of Sptlc1 in response to cocaine

**FIGURE 6:**
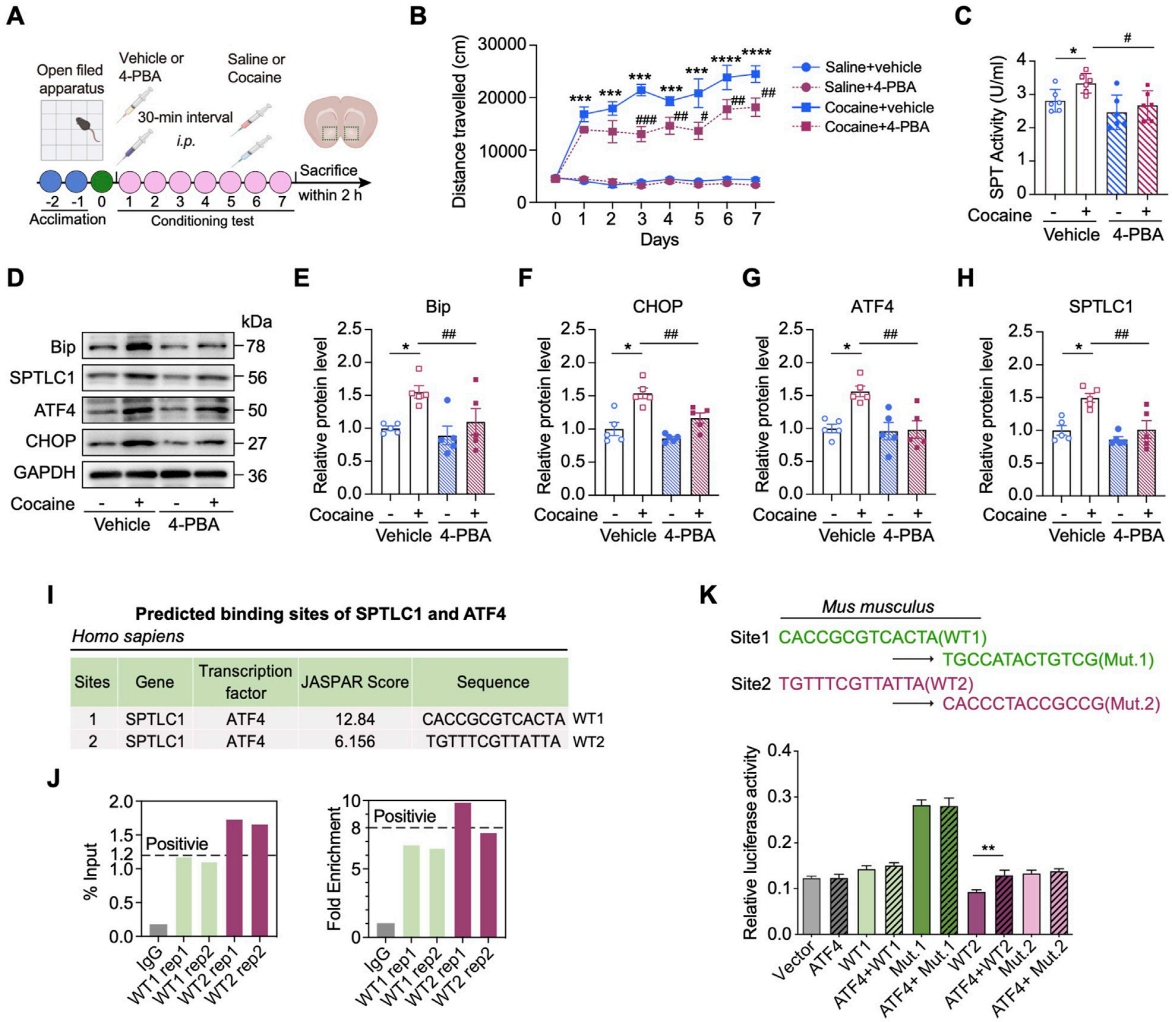
Inhibition of ER stress rescues cocaine-related neurobehaviors through ATF4‒SPTLC1 axis. **(A)** Diagram of establishing cocaine-induced hyperlocomotor activity in mice with 4-PBA pretreatment. **(B)** Total distances of mice travelled before or after **(I)**.p. injection of 20 mg/kg cocaine with 4-PBA pretreatment (90 mg/kg) and the vehicle control (n = 8). Mice received vehicle or 4-PBA 30 min before daily behavioral testing. **(C)** Bar graph shows the change of SPT activity of the NAc (n = 6). Main effects: cocaine, *F* (1,20) = 4.860, *p* = 0.0394; 4-PBA, *F* (1,20) = 9.283, *p* = 0.0064; interaction, *F* (1,20) = 0.9134, *p* = 0.3506. **(D–H)** Representative Western blot bands **(D)** and quantitative data analyses of BiP **(E)**, CHOP **(F)**, ATF4 **(G)** and SPTLC1 **(H)** (n = 5). BiP, main effects: cocaine, *F* (1,16) = 8.337, *p* = 0.0107; 4-PBA, *F* (1,16) = 4.582, *p* = 0.0481; interaction, *F* (1,16) = 1.725, *p* = 0.2076. CHOP, main effects: cocaine, *F* (1,16) = 31.25, *p* < 0.0001; 4-PBA, *F* (1,16) = 11.58, *p* = 0.0036; interaction, *F* (1,16) = 2.420, *p* = 0.1393. ATF4, main effects: cocaine, *F* (1,16) = 7.704, *p* = 0.0135; 4-PBA, *F* (1,16) = 8.560, *p* = 0.0099; interaction, *F* (1,16) = 6.553, *p* = 0.0210. SPTLC1, main effects: cocaine, *F* (1,16) = 14.28, *p* = 0.0016; 4-PBA, *F* (1,16) = 13.08, *p* = 0.0023; interaction, *F* (1,16) = 4.044, *p* = 0.0615. **(I)** In *Homo sapiens*, two putative ATF4 binding sites of promotors of *Sptlc1* predicted by the JASPAR database. Site 1 is referred to as WT1, and Site 2 as WT2. **(J)** PCR results of the ChIP products using the NAc of C57BL/6 mice. Two ATF4 binding sites (WT1, WT2) in the promoter of the mice *Sptlc1* gene correspond to those identified in humans in **I**. **(K)** Two nucleotide differences exist between the human and mouse WT1 site (highlighted in blue). The corresponding mutants, Mut.1 and Mut.2, were constructed. Dual-luciferase reporter assays were performed in HEK293 cells (n = 3) to assess the ability of ATF4 to activate the *Sptlc1* gene promoter (WT1 and WT2). For **K**, Data are presented as mean ± SEM and analyzed using unpaired two-tailed t-test. * *p* < 0.05 vs. WT2 group. For others, data are presented as mean ± SEM and analyzed using two-way ANOVA followed by Turkey’s multiple comparisons test. * *p* < 0.05; ** *p* < 0.01; *** *p* < 0.001; **** *p* < 0.0001 vs. saline group. # *p* < 0.05; ### *p* < 0.001 vs. cocaine group. ns, *p* > 0.05.

Given that cocaine induces ER stress and reprograms sphingolipid metabolism in the NAc, we hypothesized that ER stress may serve as an upstream regulator of SPTLC1 expression. To test this, we applied 4-phenylbutyric acid (4-PBA), a chemical chaperone that inhibits ER stress (Jo et al., 2019), to investigate its effects on cocaine-induced SPTLC1 upregulation and associated behavioral phenotypes. Notably, 4-PBA treatment did not alter locomotor activity in saline-treated mice but significantly attenuated the hyperlocomotion elicited by repeated cocaine administration (Figure 6A). In parallel, 4-PBA robustly suppressed cocaine-induced upregulation of SPT enzymatic activity as well as the expression of ER stress markers including BiP, CHOP, and ATF4, along with SPTLC1 (*p* < 0.01) (Figures 6B–D), suggesting that ER stress plays a causal role in driving SPTLC1 induction.

To further dissect the transcriptional mechanism underlying UPR-mediated regulation of *Sptlc1*, we utilized the UCSC genome browser and JASPAR databases to explore the transcription factors potentially, including ATF4, ATF6 and XBP1, associated with the promoter region of *Sptlc1*. Interestingly, two potential ATF4 binding sites (wild_type site 1: 1,398 bp to 1,975 bp, WT1; and wild_type site 2: 1,975 bp to 1,987 bp, WT2) in the *Sptlc1* promoter region were predicted in both the human and mouse genomes, whereas no such sites were predicted for ATF6 and XBP1 (Figure 6E). ChIP-qPCR assays confirmed ATF4 enrichment at WT2 site (Figure 6F), and luciferase assays showed WT2 (but not WT1) was essential for promoter activity (*p* < 0.01) (Figure 6G), indicating WT2 is crucial for ATF4-mediated transcriptional activation.

Together, these data indicate that, as a key transcription factor involved in ER stress, ATF4 can promote the transcriptional activity of *Sptlc1* through binding to its specific promoter region.

### 3.6 *Atf4* knockdown in D1-MSNs relieves cocaine-induced neurobehaviors

**FIGURE 7:**
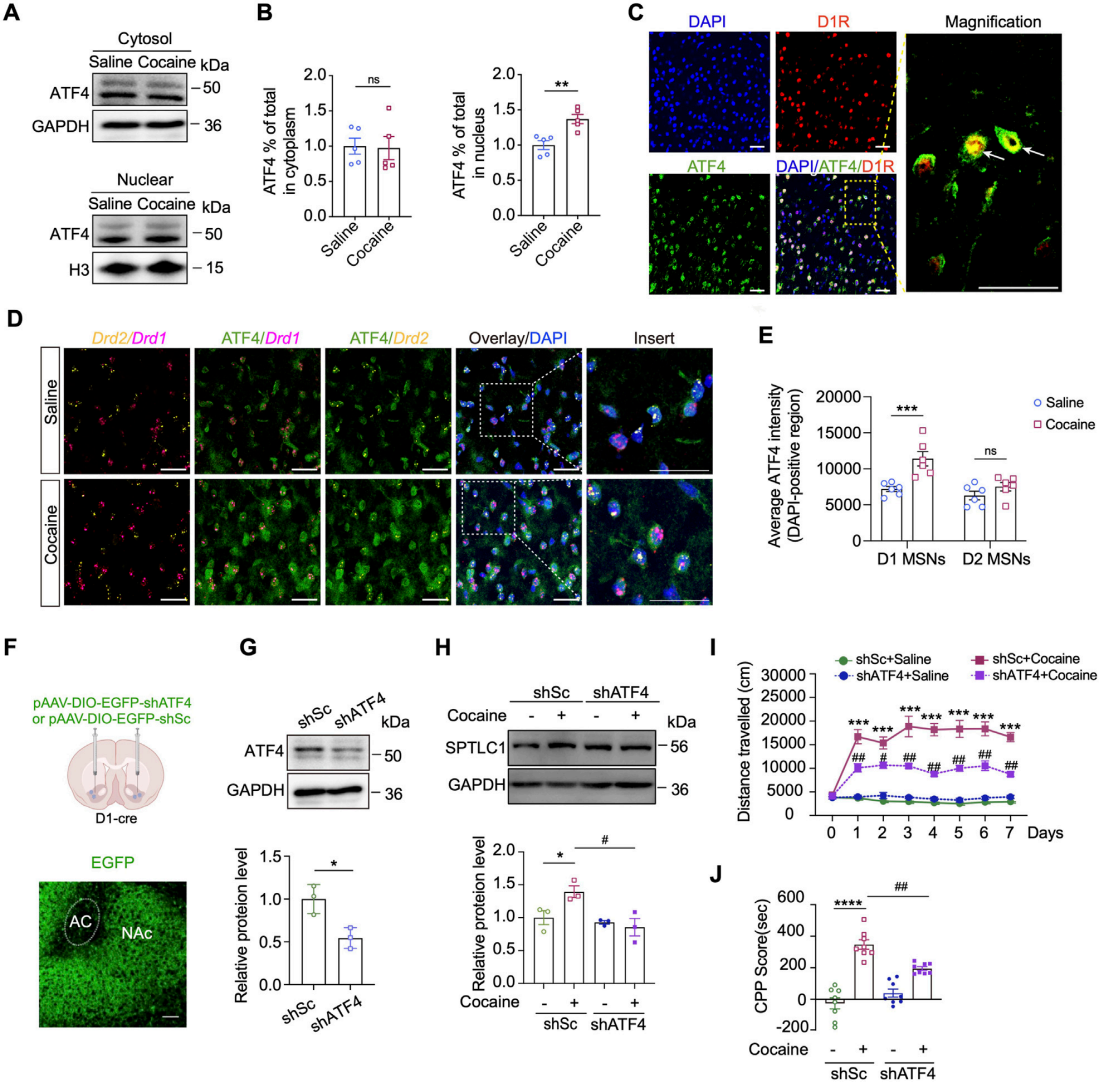
Viral-mediated knockdown of *Atf4* in the D1-MSNs represses cocaine-induced neurobehaviors. **(A,B)** Representative Western blot bands **(A)** and quantitative data analyses **(B)** of nuclear translocation of ATF4 in the NAc of mice subjected to cocaine-induced hyperlocomotion: cytosol, *t* (8) = 0.1346, *p* = 0.8963; nuclear, *t* (8) = 4.067, *p* = 0.0036 (n = 5). **(C)** Representative images of the co-immunostaining images for ATF4 and D1R in the NAc of cocaine-conditioned mice. Scale bars: 40 μm. **(D)** Representative co-immunostaining images for ATF4 (green) and RNAscope *in situ* hybridization of *Drd1* (magenta) and *Drd2* (yellow) mRNA in the NAc of mice. Scale bars: 40 μm. **(E)** Quantitative analyses of nuclear ATF4 signal in D1 and D2 MSNs. Nuclear ATF4 signal was measured within DAPI-defined nuclei using ImageJ. Six mice were analyzed, with about 150 cells quantified per group. D1-MSNs, *t* (10) = 4.103, *p* = 0.0021; D2 MSNs, *t* (10) = 1.912, *p* = 0.0850. **(F)** Upper: schematic diagram showing the microinjection of knockdown virus (AAV-shATF4) into the NAc of D1-Cre mice. Lower: representative images showing the expression of EGFP into the NAc of D1-Cre mice. Scale bars: 100 μm. **(G)** Western blot analysis and quantification of protein level of ATF4 in the NAc 3 weeks after virus administration. ATF4: *t* (4) = 3.776, *p* = 0.0195 (n = 3). **(H)** Western blot analysis and quantification of SPTLC1 expression level after *Atf4* knockdown in the NAc of mice with cocaine-induced hyperactivity (n = 3). Quantitative analysis of Western blot bands was performed by normalizing target protein intensity to GAPDH, the mean value of the shSc + saline group was set to 1. Main effects: cocaine, *F* (1,8) = 2.735, *p* = 0.1368; shATF4, *F* (1,8) = 9.9905, *p* = 0.0137; interaction, *F* (1,8) = 5.858, *p* = 0.0418. **(I)** Total distances travelled by shATF4-treated D1-Cre mice subjected to cocaine-induced hyperlocomotion (n = 8). **(J)** CPP scores of shATF4- and shSc-treated D1-Cre mice with CPP paradigm (n = 8). Main effects: cocaine, *F* (1,28) = 95.45, *p* < 0.0001; shATF4, *F* (1,28) = 2.581, *p* = 0.1194; interaction, *F* (1,28) = 16.14, *p* = 0.0004. Data are presented as mean ± SEM and analyzed using two-way ANOVA followed by Turkey’s multiple comparisons test. For **B, E and G**, data were analyzed using unpaired two-tailed *t*-test. * *p* < 0.05; ** *p* < 0.01; *** *p* < 0.001; **** *p* < 0.0001 vs. saline group. # *p* < 0.05; ### *p* < 0.001 vs. cocaine group. ns, *p* > 0.05.

To assess ATF4 activation in response to cocaine-induced ER stress, we isolated nuclear fractions from the NAc and found that repeated cocaine exposure (20 mg/kg, 7 days, i.p.) significantly increased nuclear ATF4 levels (Figures 7A,B). Dual immunofluorescence staining further confirmed enhanced nuclear translocation of ATF4 specifically in D1R-positive MSNs following cocaine administration (Figure 7C). RNAscope *in situ* hybridization combined with an immunofluorescence assay revealed the ATF4 protein (green) was specifically upregulated and translocated to the nucleus of D1-MSNs (*p* < 0.001) but not to those of D2 MSNs (*p* > 0.05) (Figures 7D,E). Together, these results demonstrate a robust and specific activation of ATF4 in D1-MSNs of the NAc in response to cocaine.

To further demonstrate that ATF4 activation is necessary for SPTLC1 upregulation, we created Cre-mediated conditional ATF4-knockdown mice with the RNAi strategy. Cre-dependent AAV-shATF4 was infused into the NAc of adult D1-Cre mice (Figure 7F). Three weeks after AAV virus injection, immunofluorescent analysis of brain slices revealed robust EGFP expression within the NAc (Figure 7F), and Western blot analysis confirmed successful knockdown of *Atf4* (*p* < 0.05) (Figure 7G). Notably, conditional *Atf4* knockdown significantly reduced SPTLC1 expression in the NAc of cocaine-conditioned mice (*p* < 0.05) (Figure 7H). We then treated *Atf4*-knockdown mice with cocaine. Results showed that *Atf4* knockdown in D1-MSNs did not affect baseline locomotor activity but effectively suppressed both cocaine-induced hyperlocomotion (Figure 7I) and cocaine-induced conditioned place preference (*p* < 0.01) (Figure 7J). Taken above, *Atf4* knockdown in D1-MSNs prevents cocaine-induced neurobehaviors by suppressing the induction of SPTLC1.

## 4 Discussion

Although the ER stress induced by psychostimulants has garnered widespread attention (Jayanthi et al., 2009; Yang et al., 2024), there is currently no conclusive evidence indicating that ER stress regulates psychostimulant-induced neuroplasticity and neurobehaviors. Here, we demonstrated that ATF4‒SPTLC1 axis in D1-MSNs is critical for cocaine-induced sphingolipid metabolism reprogramming and neurobehaviors. Cocaine specifically promotes ATF4 activation in D1-MSNs of the NAc, resulting in SPTLC1 upregulation and subsequent enhanced *de novo* sphingolipid biosynthesis. ATF4 transcriptionally regulates *Sptlc1*, and knockdown of *Sptlc1* or *Atf4* in D1-MSNs prevents cocaine effects. To our knowledge, this is the first study discovering that neuron subtype-specific modulation of ER stress and sphingolipid metabolism is a critical adaptive response to cocaine.

Studies have shown ER stress-induced neuronal death and glial cell activation through different UPR branches, whereas few have addressed the crosstalk between ER stress and neuroplasticity in the context of cocaine-elicited neurobehaviors (Barrientos et al., 2018). Our findings discovered cocaine-induced ER stress along with the activation of the eIF2α‒ATF4 pathway specifically in D1-MSNs of the NAc. The eIF2α-ATF4 pathway is known to mediate an immediate adaptive reaction to ER stress (Balsa et al., 2019), and ATF4 expression selectively promotes the transcription of downstream genes that respond to ER stress (Vasudevan et al., 2020), such as CHOP and BiP. Consistent with our findings, psychostimulant exposure has been reported to induce ATF-family transcription factors and translational remodeling in the striatum *via* dopamine receptor–dependent mechanisms (Green et al., 2008; Beauvais et al., 2011; Biever et al., 2017). These results collectively suggest that activation of the eIF2α–ATF4 branch of the UPR represents a conserved neuroadaptive response coupling dopaminergic signaling to translational control and synaptic plasticity during psychostimulant-induced stress. Moreover, pharmacological inhibition of this UPR pathway by 4-PBA attenuated cocaine-induced molecular and behavioral changes, indicating that ER stress constitutes a critical adaptive mechanism underlying cocaine-elicited neuroplasticity. In this study, both immunofluorescence and RNAscope analysis revealed predominant activation of ATF4, specifically in D1-MSNs, after cocaine exposure. Furthermore, silencing of the gene encoding ATF4 in D1-MSNs significantly inhibited cocaine-induced neurobehaviors. These findings reveal a critical role for ATF4 activation in D1-MSNs in mediating the effects of cocaine.

In neurons, sphingolipid metabolism is precisely regulated to ensure sufficient sphingolipid synthesis to meet the requirements for membrane remodeling and synaptic plasticity (Crivelli et al., 2020; Goyal et al., 2019). In our study, we observed distinct sphingolipid alterations in the NAc under both the cocaine-induced locomotor activity and CPP paradigms, with CPP eliciting more pronounced changes. These differences may stem from both the distinct cocaine administration schedules and the timing of tissue collection. In the hyperlocomotion paradigm, mice received daily cocaine injections for seven consecutive days, and tissues were collected within 2 h after the last exposure. At this time point, animals may have already adapted to the repeated cocaine stimulation. In contrast, in the CPP paradigm, mice underwent three intermittent cocaine exposures and tissues were collected 24 h after the final conditioning, corresponding to a phase when associative learning and memory consolidation are engaged. This likely imposes higher demands on membrane remodeling and synaptic plasticity, processes that require dynamic sphingolipid metabolism. Supporting this, previous animal studies reporting changes in brain sphingolipid levels during drug exposure (Frankowska et al., 2021; Kalinichenko et al., 2018), and clinical evidence showed that sphingolipid accumulation in the peripheral serum or hair may be a reliable indicator of drug addiction (Liu et al., 2022; Kim et al., 2020). However, there is little evidence clarifying the role of metabolic enzymes in reprogramming sphingolipid metabolism during cocaine treatment. Through screening the mRNA levels of various enzymes involved in sphingolipid metabolism, we observed pronounced expression of *Sptlc1* in D1-MSNs. Interestingly, cocaine specifically and markedly upregulated SPTLC1 in the NAc. Although SPTLC1 is expressed throughout the body, particularly in the liver, we did not observe a change in SPTLC1 expression in the liver or other brain regions in response to cocaine. More importantly, gene silencing of *Sptlc1* in D1-MSNs significantly mitigated cocaine-mediated behaviors. Together, these results show that SPTLC1 upregulation in the D1-MSNs is necessary for the formation of cocaine-induced neurobehaviors. Due to limitations associated with available D1R and D2R antibodies, including variability in signal intensity and occasional background staining, precise assignment of SPTLC1 to individual MSN subtypes can be challenging. While our results support predominant localization of SPTLC1 in D1-MSNs, Future studies using MSN subtype-specific fluorescent reporters or single-cell RNA sequencing combined with immunostaining may provide a more precise assessment of SPTLC1 distribution across neuronal subtypes.

Drug-induced adaptive alterations in the structural and physiological properties of dendritic spines are crucial for long-term and compulsive drug-seeking behaviors (Barrientos et al., 2018; Cahill et al., 2018). Consistently, we showed that cocaine markedly increased the dendritic complexity of MSNs in the NAc, as evidenced by increased dendritic length, dendritic bifurcation, and spine density. Cocaine exposure leads to the formation of new synapses (synaptogenesis), which are believed to mature into fully functional synapses that play a critical role in the consolidation of cocaine-associated memories (Zinsmaier et al., 2022). These changes in synaptic architecture significantly contribute to the persistence of cocaine induced behavioral changes. We showed that *Sptlc1* knockdown in D1-MSNs effectively attenuated cocaine-induced dendritic remodeling and neurobehavioral effects. Notably, *Sptlc1* knockdown also affect neuronal morphology even under saline conditions, particularly at distal regions from the soma, suggesting that SPTLC1 may play a role in maintaining basal neuronal structure independent of cocaine exposure. As a key enzyme in sphingolipid biosynthesis, SPTLC1 is critical for normal neuronal architecture, since sphingolipids are essential components of neuronal membranes. However, the specific molecular mechanism by which sphingolipids mediate D1-MSNs structural plasticity remains unexplored. Future research, including targeted metabolic flux assays and sphingolipid synaptic trafficking analysis, is needed to address these issues.

How does ER stress regulate SPTLC1 expression in the development of cocaine induced behavioral changes? It was reported that ER stress can induce the upregulation of SPTLC2 and then reprogram sphingolipid metabolism in the liver (Kim et al., 2022). Interestingly, the transcript level of *Sptlc1*, but not that of *Sptlc2/3*, was increased in the NAc by cocaine. We first demonstrated that ATF4 is a transcription factor that directly binds to the promoter of *Sptlc1*. Pharmacological inhibition of ER stress or knockdown of *Atf4* in the NAc of D1-Cre mice reduced the expression of SPTLC1. Both ChIP and luciferase assays unambiguously justified the role of ATF4 as a transcription factor and provided compelling evidence that ATF4 binds to the promoter of *Sptlc1*. Moreover, the eIF2α‒ATF4 pathway was activated more strongly than the XBP1–ATF6 pathway, suggesting that activation of the eIF2α‒ATF4 branch of the UPR is mechanistically contributes to ER stress-induced SPTLC1 upregulation. It is important to note that while SPTLC1 typically forms a complex with SPTLC2 and SPTLC3, the regulation of SPTLC1 can occur independently under certain conditions. Although SPTLC1 itself lacks intrinsic catalytic activity, elevated SPTLC1 expression can stabilize the SPT complex and enhance sphingolipid synthesis under ER stress (Lone et al., 2020). In addition to SPTLC1, previous studies have shown that ATF4 can transcriptionally activate SPHK1 involved in sphingolipid metabolism (Lan et al., 2024). Given that SPTLC1 catalyzes the first and rate-limiting step in sphingolipid biosynthesis, its regulation likely represents a key driver of the lipid remodeling observed here, although ATF4 may also influence other metabolic targets.

It has been known that D1 and D2 MSNs show different expression patterns and downstream signaling pathways (Kruyer et al., 2021; Tran et al., 2021). Although we did not explore the mechanism underlying D1 MSN-specific activation of the ATF4‒SPTLC1 axis, several hypotheses warrant further investigation: 1) Differences in downstream signaling pathways: for instance, cocaine could increase extracellular dopamine by blocking the dopamine transporter (DAT), thereby enhancing D1R activation and downstream cAMP/PKA signaling, which may increase the phosphorylation of the elF2α and ATF4 (Soda et al., 2013; Rajesh et al., 2008); 2) Regulatory mechanisms: D1 and D2 MSNs might employ distinct mechanisms in regulating ER homeostasis and stress responses, such as differences in the expression levels of ER chaperones or the efficiency of the protein folding machinery (Free et al., 2007); 3) Differential gene expression: D1 and D2 MSNs may express different arrays of genes involved in the ER stress response pathways, potentially rendering D1-MSNs more susceptible to ER stress (Tran et al., 2021). Recently, cell type-specific transcriptomic sequencing revealed that after cocaine exposure, a set of genes involved in the cellular stress response were upregulated in D1-MSNs in the NAc of mice (Mews et al., 2023). This result further supports our findings in discovering the contribution of D1-MSNs in mediating the effects of cocaine through an integrated ER stress response.

## 5 Conclusion

In conclusion, our findings reveal that in the context of cocaine exposure, ER stress‒mediated ATF4 signaling activation modulates *de novo* sphingolipid biosynthesis in D1-MSNs of the NAc. This pathway, mediated by selective upregulation of SPTLC1, is critical for cocaine-induced behaviors and neuroplasticity. These findings provide a novel therapeutic strategy for cocaine addiction through targeting ER stress and ATF4‒SPTLC1 axis.

## Data Availability

The data presented in the study are deposited in the iProX repository, accession number IPX0014284000.

## Abbreviations

ER, endoplasmic reticulum; MSNs, medium spiny neurons; NAc, nucleus accumbens; D1R, dopamine receptor 1; SPTLC1, serine palmitoyltransferase long-chain base subunit 1; SPT, serine palmitoyltransferase; ATF4, activating transcription factor 4; UPR, unfolded protein response; CPP, conditioned place preference; XBP1, X-box binding protein 1; CHOP, DNA damage-inducible transcript 3; BiP, binding immunoglobulin protein; eIF2α, eukaryotic translation initiation factor 2 alpha; ELISA, enzyme-linked immunosorbent assay; SPH, sphingosine; SM, sphingomyelin; Cers, ceramide synthase; Asah1, N-acylsphingosine amidohydrolase 1; Sgms1, sphingomyelin synthase 1; Sphk2, sphingosine kinase 2.
