# Supplementary material for: ER stress in D1-MSNs mediates cocaine-induced behavioral plasticity via the ATF4–SPTLC1 axis
Source: Front Pharmacol. 2025 Nov 25;16:1677343. doi: 10.3389/fphar.2025.1677343 (PMC12685851; doi:10.3389/fphar.2025.1677343)
Supplement: Supplementary file 1 [file Supplementaryfile1.docx]

Supplementary Material

# Supplementary Figure1


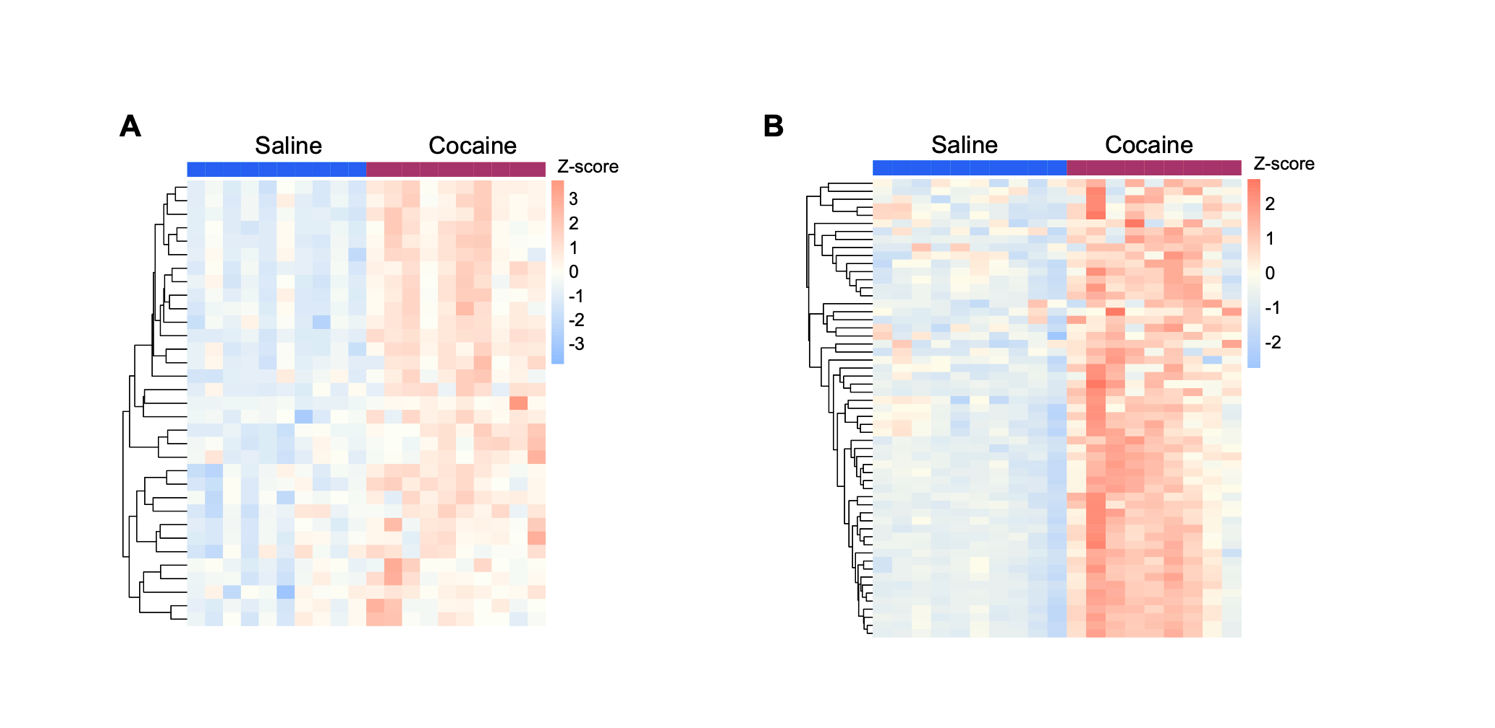


**Supplementary Figure 1. Heatmap visualization of upregulated lipids in cocaine-treated mice.** (**A)** Heatmap showing the relative abundance of significantly upregulated lipid species in the brain of mice subjected to the cocaine-induced hyperlocomotion model (n = 10). (**B)** Heatmap showing the relative abundance of significantly upregulated lipid species in the brain of mice subjected to the cocaine-induced conditioned place preference (CPP) paradigm (n = 10 for saline group and n=9 for cocaine group). For both datasets, lipids were z-score normalized across samples, and hierarchical clustering was applied to both rows (lipid species). Warmer colors indicate higher relative abundance, and cooler colors indicate lower abundance.

# Supplementary Figure2

**
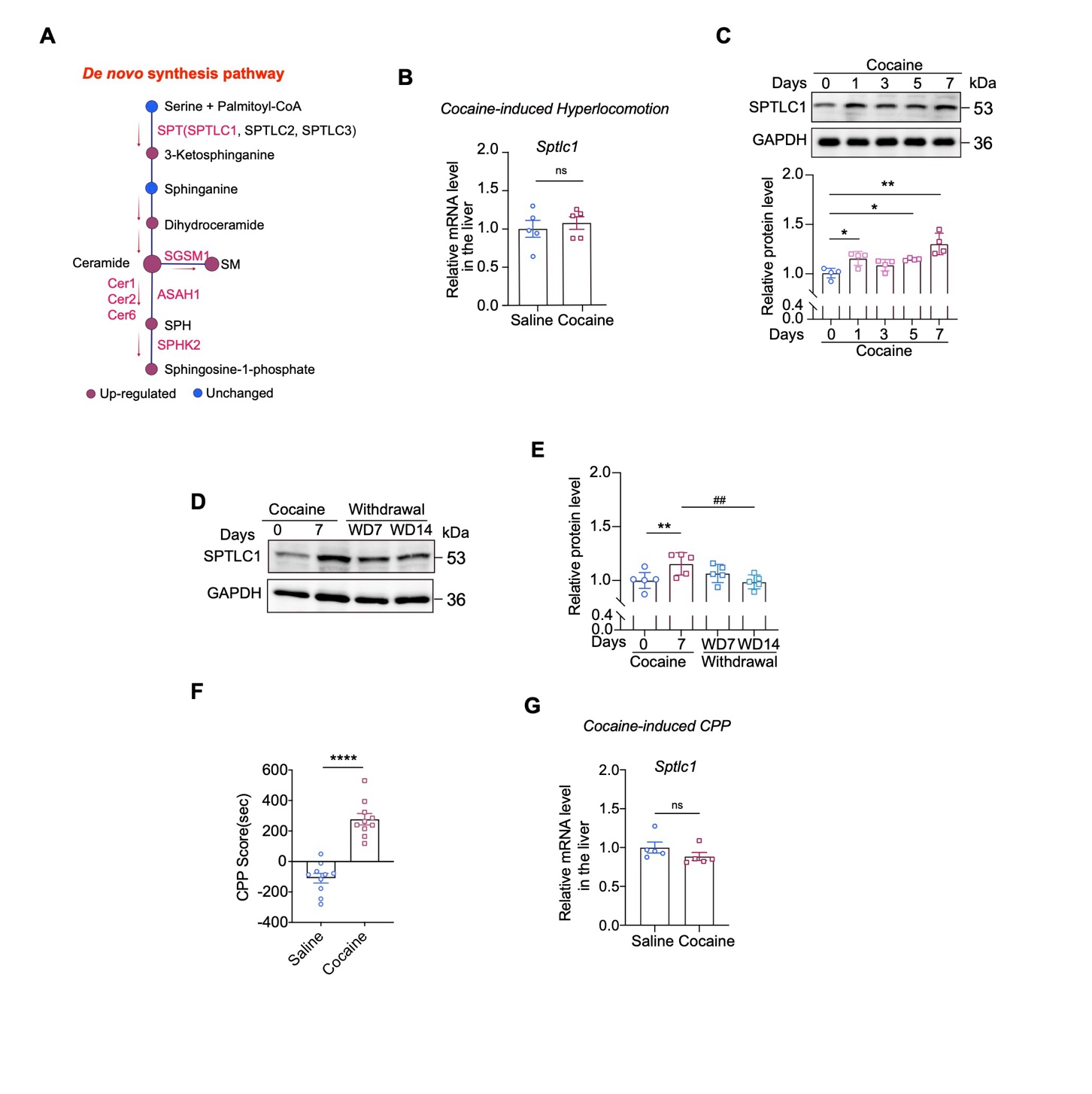
**

**Supplementary Figure 2. Cocaine specifically induces SPTLC1 expression in the NAc**

(**A)** Schematic representation of alterations in the sphingolipid metabolism pathway in the NAc of mice with cocaine-induced hyperlocomotion. Colors indicate the direction of change for each lipid class (increased or decreased), while the relative size of each circle reflects the total abundance of the corresponding lipid class, the relevant enzymatic nodes were also noted. (**B)** Expression of *Sptlc1* genes measured by qPCR in liver of mice subjected to cocaine-induced hyperlocomotion (n = 5). **(C)** Representative western blot images and quantification of SPTLC1 protein levels in the NAc, collected 2 hours after the last daily cocaine injection (20 mg/kg) for 7 days (n = 4). **(D, E)** Western blot bands and quantification of SPTLC1 in the NAc of mice treated daily with cocaine for 7 consecutive days, followed by withdrawal periods of 7 or 14 days (WD7, WD14). NAc tissue was collected within 2 hours of the last cocaine injection (day 7) and at 7 or 14 days after cessation of cocaine administration (n = 5). (**F)** CPP score of mice from cocaine-induced CPP model (n = 10). (**G)** Expression of *Sptlc1* genes measured by qPCR in the liver of cocaine CPP mice (n = 5). All qPCR values were normalized to GAPDH, and western blot signals were normalized to GAPDH. For both qPCR and western blot, the saline group was set to 1 and all other groups are expressed as fold change relative to saline. Data are presented as mean ± SEM. * *p* < 0.05, ** *p* < 0.01, *** *p* < 0.001 by unpaired two-tailed *t* test *vs* saline group. ^##^p < 0.01 by unpaired two-tailed *t* test *vs* cocaine (7 day) group. ns, *p* > 0.05.

# Supplementary Figure3


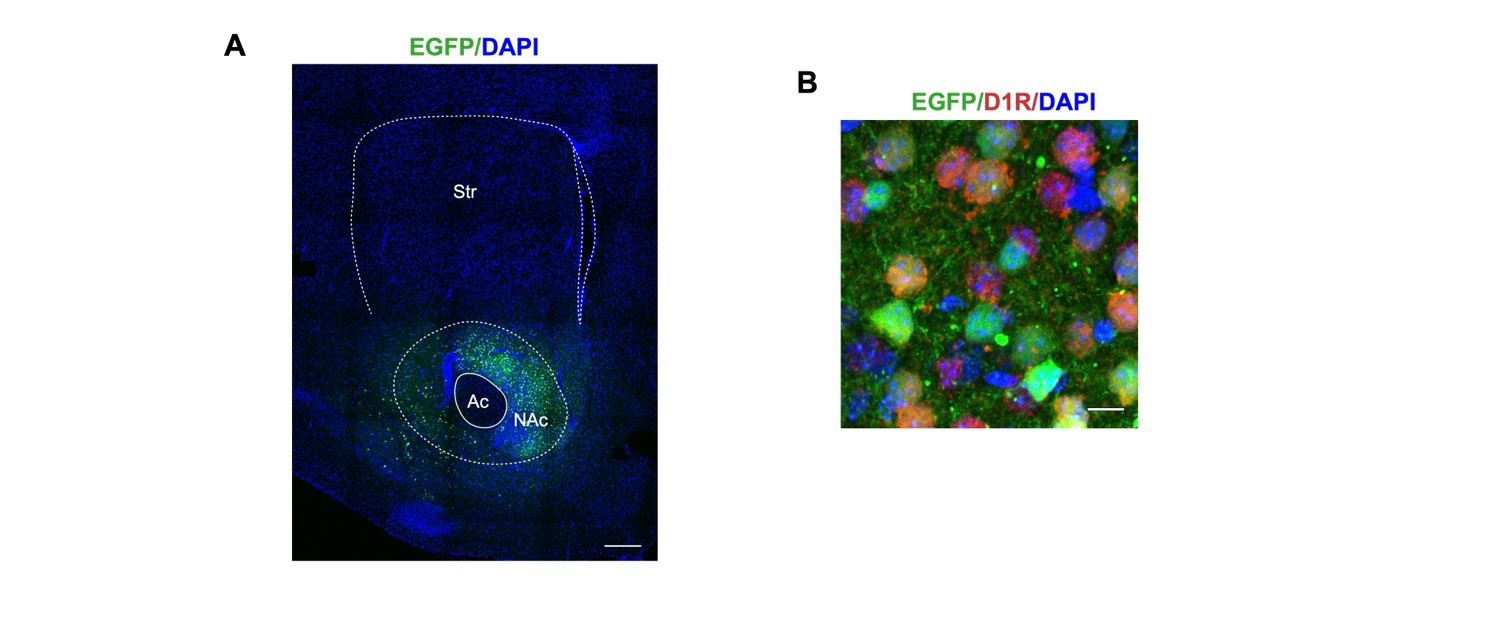


**Supplementary Figure 3. Virus injection and infection specificity in the NAc**

(**A)** Representative fluorescence image showing EGFP expression in the NAc of D1-Cre mice following AAV-DIO injection. Scale bar: 250 μm. (**B)** Co-immunofluorescence of viral EGFP signal with D1R antibody labeling, confirming infection specificity within D1-MSNs. Scale bar: 10 μm.

# Supplementary Table 1

qPCR primers **(***Mus musculus***)** used in the study

| **qPCR primers** | **Forward** | **Reverse** |
| --- | --- | --- |
| *Sptlc1* | TGTGTAGTAGGTTCGGAAGCAAAGTG | GGCAGGTGAGAGGAATGAGACAATG |
| *Sptlc2* | CAACTATCTTGGATTTGCGAGG | CTTGTCCAGGTTTCCAATTTCC |
| *Sptlc3* | CAAAGGACAGATCGTGGAAACAGAAAC | CGTGAGGTGCTGAGAAGAAGGTATATC |
| *GAPDH* | AGGTCGGTGTGAACGGATTTG | GAGTTGCTGTTGAAGTCGCA |
| *Cers1* | CCACCACACACATCTTTCGG | GGAGCAGGTAAGCGCAGTAG |
| *Cers2* | ATGCTCCAGACCTTGTATGACT | CTGAGGCTTTGGCATAGACAC |
| *Cers6* | GATTCATAGCCAAACCATGTGCC | AATGCTCCGAACATCCCAGTC |
| *Sgms1* | TTGGCACGCTGTACCTGTATC | CAGTCTCCAAAGAGCTTCGGA |
| *Asah1* | CGTGGACAGAAGATTGCAGAA | TGGTGCCTTTTGAGCCAATAAT |
| *Sphk2* | CACGGCGAGTTTGGTTCCTA | CTTCTGGCTTTGGGCGTAGT |
| *XBP1s* | GCTGAGTECGCAGCAGGT | CAGGGTCCAACTTGTCAGAAT |
| *Ddit* | CTGGAAGCCTGGTATGAGGAT | CTGGAAGCCTGGTATGAGGAT |
| *WT1* | CAAGCACTTCACACCCGAAC | CGTGTCAAATAGTGCGCGG |
| *WT2* | ATTGCGTGTTACTTAAAAGGGA | CCCTGCCTCGACAAAACTA |
